# Activation of the cardiac non-neuronal cholinergic system prevents the development of diabetes-associated cardiovascular complications

**DOI:** 10.1186/s12933-021-01231-8

**Published:** 2021-02-22

**Authors:** Eng Leng Saw, James T. Pearson, Daryl O. Schwenke, Pujika Emani Munasinghe, Hirotsugu Tsuchimochi, Shruti Rawal, Sean Coffey, Philip Davis, Richard Bunton, Isabelle Van Hout, Yuko Kai, Michael J. A. Williams, Yoshihiko Kakinuma, Martin Fronius, Rajesh Katare

**Affiliations:** 1 Department of Physiology, HeartOtago, School of Biomedical Sciences, University of Otago, 270, Great King Street, Dunedin, 9016 New Zealand; 2 Department of Cardiac Physiology, National Cerebral and Cardiovascular Center Research Institute, Suita, Japan; 3 Biomedicine Discovery Institute and Department of Physiology, Monash University, Melbourne, VIC Australia; 4 Department of Medicine, School of Medicine, University of Otago, Dunedin, New Zealand; 5 Department of Cardiothoracic Surgery, School of Medicine, University of Otago, Dunedin, New Zealand; 6 Department of Bioregulatory Science, Graduate School of Medicine, Nippon Medical School, Tokyo, Japan

**Keywords:** Non-neuronal cholinergic system, Acetylcholine, Diabetic heart disease, Glucose metabolism, Angiogenesis

## Abstract

**Background:**

Acetylcholine (ACh) plays a crucial role in the function of the heart. Recent evidence suggests that cardiomyocytes possess a non-neuronal cholinergic system (NNCS) that comprises of choline acetyltransferase (ChAT), choline transporter 1 (CHT1), vesicular acetylcholine transporter (VAChT), acetylcholinesterase (AChE) and type-2 muscarinic ACh receptors (M_2_AChR) to synthesize, release, degrade ACh as well as for ACh to transduce a signal. NNCS is linked to cardiac cell survival, angiogenesis and glucose metabolism. Impairment of these functions are hallmarks of diabetic heart disease (DHD). The role of the NNCS in DHD is unknown. The aim of this study was to examine the effect of diabetes on cardiac NNCS and determine if activation of cardiac NNCS is beneficial to the diabetic heart.

**Methods:**

Ventricular samples from type-2 diabetic humans and db/db mice were used to measure the expression pattern of NNCS components (ChAT, CHT1, VAChT, AChE and M_2_AChR) and glucose transporter-4 (GLUT-4) by western blot analysis. To determine the function of the cardiac NNCS in the diabetic heart, a db/db mouse model with cardiac-specific overexpression of *ChAT* gene was generated (db/db-ChAT-tg). Animals were followed up serially and samples collected at different time points for molecular and histological analysis of cardiac NNCS components and prosurvival and proangiogenic signaling pathways.

**Results:**

Immunoblot analysis revealed alterations in the components of cardiac NNCS and GLUT-4 in the type-2 diabetic human and db/db mouse hearts. Interestingly, the dysregulation of cardiac NNCS was followed by the downregulation of GLUT-4 in the db/db mouse heart. Db/db-ChAT-tg mice exhibited preserved cardiac and vascular function in comparison to db/db mice. The improved function was associated with increased cardiac ACh and glucose content, sustained angiogenesis and reduced fibrosis. These beneficial effects were associated with upregulation of the PI3K/Akt/HIF1α signaling pathway, and increased expression of its downstream targets—GLUT-4 and VEGF-A.

**Conclusion:**

We provide the first evidence for dysregulation of the cardiac NNCS in DHD. Increased cardiac ACh is beneficial and a potential new therapeutic strategy to prevent or delay the development of DHD.

## Background

Diabetic heart disease (DHD) is a common morbidity and the leading cause of mortality among people with diabetes, constituting more than 80% of deaths [1–3]. Importantly, this high mortality is observed despite the marked advances in current health care systems and improved management of diabetes. In type-2 diabetes mellitus (T2DM), insulin resistance and metabolic derangements suppress glucose metabolism in the heart due to downregulation of glucose transporter-4 (GLUT-4) expression [4–7]. With the progression of T2DM, prolonged exposure to metabolic derangements contributes to endothelial dysfunction, leading to coronary artery disease (CAD) and coronary microvascular disease (CMVD) [8–10]. CAD and CMVD reduce coronary artery blood flow and myocardial perfusion, which increases the workload on the cardiac muscle [11] and contributes to the development of DHD.

Previous studies demonstrated that cardiomyocytes possess a functional intrinsic cholinergic machinery, known as the non-neuronal cholinergic system (NNCS). The NNCS in the cardiomyocytes comprises different components to maintain homeostasis of acetylcholine (ACh) as well as to allow ACh to act as an auto-/paracrine mediator (reviewed in [12]). These components are choline acetyltransferase (ChAT) to synthesize ACh; choline transporter1 (CHT1) for the reuptake of choline into the cardiomyocytes for ACh synthesis; vesicular ACh transporter (VAChT) to store and release ACh; acetylcholinesterase (AChE) to degrade ACh in the extracellular space as well as type-2 muscarinic ACh receptor (M_2_AChR) for ACh binding and signal transduction [13–15]. We, along with others, have reported the crucial role of ACh from cardiomyocytes for the maintenance of glucose and cellular homeostasis [16–18] and as a promoter of angiogenesis [18–20] in the heart. The ACh released from cardiomyocytes acts as an auto-/paracrine mediator. Its binding to M_2_AChR initiates activation of pro-survival phosphatidylinositol-3-kinase (PI3K)/protein kinase B (Akt)/hypoxia-inducible factor 1-alpha (HIF1α) signaling cascade [18, 19, 21]. Activation of this cascade was reported to upregulate the expression of downstream targets including: (1) glucose transporter-4 (GLUT-4) to promote glucose uptake and energy preservation [16–18, 22, 23], and, (2) vascular endothelial growth factor (VEGF) to promote angiogenesis [18–21]. However, to the best of our knowledge, the involvement of cardiac NNCS in the pathophysiology of DHD is unknown. This is of particular importance as diabetes is associated with impairment of glucose homeostasis, cell survival and angiogenesis.

The aim of the current study was to determine whether the cardiac NNCS is affected in diabetes and to reveal if its modulation is beneficial in preventing the development and progression of DHD. To address the first part of our aim, left ventricular tissue samples from T2DM human and diabetic (db/db) mice were used to compare expression levels of cardiac NNCS components with those from non-diabetic samples. For the second part of our aim, a transgenic type 2 diabetic db/db mouse model, characterized by overexpression of the *ChAT* gene in cardiomyocytes (db/db-ChAT-tg), was used to study cardiac and vascular function in comparison to db/db mice. Overall, the results revealed, differences in NNCS components in diabetic hearts (human and mice) and sustained cardiac and vascular function and reduced cardiac fibrosis in db/db-ChAT-tg mice compared to the db/db mice. Our data suggest that activation of cardiac NNCS, characterized by increased ACh synthesis in the heart is beneficial to the diabetic heart.

## Materials and methods

The data that support the findings of this study are available from the corresponding authors upon reasonable request.

See Additional file 1 for detailed methods and regulatory approval.

### Ethical approval

The Animal Ethics Committee from the University of Otago (AEC25/12), Nippon Medical School (27-003) and the Animal Experiment Committee of SPring-8 (2018A1282), which conform to the guidelines from Directive 2010/63/EU of the European Parliament on the protection of animals used for scientific purposes approved the use of animals in this study. The Health and Disability Ethics Committee in New Zealand approved the use of human left ventricular (LV) tissues (LRS/12/01/001/AM13). The human LV tissues of patients with or without type-2 diabetes with coronary artery disease (CAD) who underwent coronary artery bypass grafting (CABG) surgery in Dunedin hospital were collected following written informed consent. The investigation with human tissue conformed to the principles outlined in the Declaration of Helsinki.

### Type-2 diabetic human left ventricular tissues

Human LV tissues were collected from type-2 diabetic (D-CAD) and non-diabetic (ND-CAD) patients with CAD undergoing on-pump coronary artery bypass graft surgery at Dunedin Hospital. The selection criteria of diabetic patients were glycated hemoglobin (HbA1c) level ≥ 50 nmol/mol and diabetes duration of more than 1 year [24]. STROBE recommendations for reporting case–control studies are available as Additional file 1: Table SI.

### Animal models

Diabetic C57BL/ksJ-lepr + / + (db/db) and their non-diabetic (ND) C57BL/ksJ-lepr + littermates (db/ +) (Jackson Laboratory) were used as the model of type-2 diabetes and age-matched controls, respectively [2, 3]. At the end of the study, mice were anaesthetized with 5% isoflurane inhalation until the breathing stopped and euthanasia was performed by excision of the heart. The ventricular tissues of db/db and ND db/ + mice collected at 12–16 weeks (early stage), 20–24 weeks (established stage) and 28–32 weeks (progressed stage) of age were used for western blot analyses.

The mouse strain with cardiac-specific overexpression of murine *ChAT* gene (ChAT-tg) was previously reported as a model of activated cardiac NNCS [18]. These ChAT-tg mice (derived from C57BL6/J background) were crossbred with the heterozygous db/ + mice to generate heterozygous db/ + mice with ChAT transgene. The heterozygous littermates were further crossbred to generate homozygous db/db mice with cardiac-specific ChAT transgene (db/db-ChAT-tg). Diabetes was confirmed by measuring blood glucose level.

### Measurement of cardiac ACh level

ACh was isolated and purified from the LV of ND, db/db and db/db-ChAT-tg mice as previously described [18]. Ten microliters of purified ACh sample was injected into an HPLC system (HTEC-500; Eicom, Japan). The results are expressed as nmol/g.

### Measurement of cardiac glucose level

Cardiac glucose level was determined from the LV tissue of ND-CAD and D-CAD patients using the Abcam glucose assay kit (AB65333) and ND, db/db and db/db-ChAT-tg mice using BioVision glucose assay kit (K606-100). Absorbance was measured at 570 nm by SpectraMax i3x microplate reader (Molecular Device, USA. The results were expressed as nmol/g. Both kits have the same sensitivity to detect glucose in the range of 1–10,000 μM and same experimental procedures.

### Evaluation of cardiac function using a pressure–volume (PV) catheter

Cardiac function was measured in the db/db-ChAT-tg and db/db mice after anesthetizing the mice with isoflurane (1–2%) at a flow rate of 0.5–1.0 L/min, as described earlier [25]. Hemodynamic parameters were measured using LabScribe2 software (iWorx, USA).

### Synchrotron radiation coronary microangiography

This experiment was performed to visualize and measure in-vivo coronary function. The experiment was performed at the SPring-8 facility, BL28B2 beamline, Hyogo, Japan, using established protocols from our previous study in both db/db-ChAT-tg and db/db mice (early and established stage of diabetes) [10]. The study was blinded by generating a unique number for each mouse to avoid bias during the analysis. The mice were anesthetized with isoflurane (1.5–5%) at a flow rate of 1L/min and positioned on a heating pad. Following a baseline angiogram, the endothelium-dependent vasodilatory response to ACh and endothelium-independent vasodilatory response to sodium nitroprusside (SNP) were assessed sequentially. The total numbers of first, second, third, and fourth-order coronary vessels on the angiogram was counted (Fig. 5a, see below). Since the fourth-order coronary vessels were only visualized in a small number of mice, the number of fourth-order coronary vessels was combined with the number of third-order coronary vessels for analysis. Detailed method and protocol are available in the Additional file 1.

### Protein extraction and western blot analysis

Proteins were extracted from mouse and human LV samples and used for western blotting as described previously [2]. Details of the primary and secondary antibodies used are provided in the Additional file 1. The chemiluminescence signal to visualize the proteins was captured by a Syngene Pxi imaging system (USA). The full blot for each antibody is shown in the Additional file 1. Band density was analyzed using Image J software (NIH). The target protein was normalized to the band intensity of a prominent band between 37 and 50 kDa from Ponceau S stained blot (total protein expression level) [26] and expressed as fold changes relative to the control group [27].

### Immunohistochemistry analysis

For detection of ChAT, GLUT-4 and cardiac troponin I (cTnI, a cardiac marker), seven micrometer thick sections were probed with anti-ChAT (Abcam, AB181023), anti-GLUT-4 (NovusBio, NBP1-49533) and biotin-conjugated anti-cTnI antibodies (NovusBio, NB110-2546B) in a sequential manner.

For microvascular analysis, seven micrometer thick sections were probed with biotin-conjugated Isolectin-B4 (Vector laboratories, B1205; 1:200) and anti-α-smooth muscle actin conjugated with Cy3™ (Sigma-Aldrich, C6198; 1:800) to detect endothelial cells and smooth muscle cells, respectively. The vascular density was expressed as the mean number of Isolectin^+^ cells (for capillaries) or αSMA^+^ and Isolectin^+^ cells (for arterioles) per mm^2^ of cardiac tissue.

To determine the level of fibrosis, sections were stained with 0.1% Direct Red 80 (Sigma Aldrich, 3,665,548)/Picric acid solution (Sigma Aldrich, 197,378). The fibrotic area was normalized to total tissue area and expressed as fold change relative to the control group.

### Statistical analysis

All statistical analyses were performed using GraphPad Prism (version 8). Shapiro–Wilk test was used to test the normality. A non-parametric Mann–Whitney U test (two-group comparison) or Kruskal–Wallis test with Bunn’s test (three groups comparison) was used to analyze the western blot data from the animal study. Unpaired T-test was used to analyze the western blot data and the clinical indices of patients. Two-way ANOVA, followed by Sidak’s test for multiple comparison test, was performed to compare body weight, cardiac glucose content and cardiac functions. A non-parametric Mann–Whitney U test was used to analyze the number of coronary vessels, percentage changes of diameter in response to ACh and SNP, the density of small, large arterioles, total capillaries as well as fibrotic area. Unpaired T-test was used to analyze the cardiac glucose content. Data are expressed as the mean ± standard error of the mean (SEM). A p-value < 0.05 was considered as statistically significant.

## Results

The representative complete blots of all the protein targets are included in Additional file 1.

### Diabetes-induced downregulation of cardiac NNCS

Our first aim was to reveal if the cardiac NNCS is changed in the heart of patients with diabetes and if this is associated with changes in cardiac glucose content.

Table 1 reports the metabolic profile, hemodynamic parameters, cardiac function, and LV structural profile of ND-CAD and D-CAD patients whose samples were used in this study. D-CAD patients had increased BMI (p < 0.05) and elevated glycated hemoglobin levels (HbA1c, p < 0.0001) compared to ND-CAD patients. D-CAD patients also had diastolic and systolic LV dysfunction as indicated by a significantly decreased E/A ratio and ejection fraction (EF). Most of the patients in this study were treated with statins, beta-blockers, ACE inhibitors and calcium channel blockers. Additionally, D-CAD patients were treated with anti-diabetic agents such as metformin, insulin and sulfonylurea to manage the blood glucose level (Table 1).

**Table 1:** Patient characteristics

	ND-CAD (n = 27)	D-CAD (n = 20)	p-value
Age (year)	68.7 ± 1.6	69.2 ± 1.9	0.8
Gender (male/female)	24/3	13/7	
Metabolic profile			
BMI (kg/m^2^)	29.3 ± 0.9	33.9 ± 1.1	0.002**
Diabetic duration (years)	–	11.3 ± 1.7	
HbA1c (mmol/mol)	37.1 ± 0.7	55.3 ± 2.5	< 0.001***
Hemodynamic profile			
MAP (mm Hg)	95.0 ± 2.6	95.9± 2.6	0.81
LVEDV (mL)	102.0 ± 7.9	106.8 ± 9.9	0.74
LVESV (mL)	47.6 ± 5.7	59.0 ± 8.5	0.29
HR (beats/min)	73.9 ± 4.2	71.7 ± 2.6	0.68
SV (mL)	54.4 ± 3.0	47.7 ± 3.2	0.22
CO (L/min)	4.0 ± 0.4	3.5 ± 0.2	0.4
Diastolic profile			
E/A	1.0 ± 0.1	0.7 ± 0.05	0.03*
E/e’	11.6 ± 0.7	13.1 ± 1.0	0.21
decT (msec)	245.8 ± 11.9	271.8 ± 19.8	0.24
Systolic profile			
Ejection fraction (%)	55.3 ± 1.8	49.1 ± 2.0	0.027*
Fractional shortening (%)	30.6 ± 1.2	30.3 ± 2.6	0.93
Ventricular structure			
IVSd (cm)	1.2 ± 0.1	1.3 ± 0.1	0.56
LVPWd (cm)	1.1 ± 0.04	1.0 ± 0.1	0.66
LVIDd (cm)	4.6 ± 0.1	4.9 ± 0.2	0.21
LVIDs (cm)	3.2 ± 0.1	3.4 ± 0.2	0.34
Medications			
Statins	21/27 (77.8%)	18/20 (90.0%)	
Beta-blockers	20/27 (74.1%)	15/20 (75.0%)	
ACE inhibitors	11/27 (40.7%)	12/20 (60.0%)	
Calcium channel blockers	3/27 (11.1%)	14/20 (70.0%)	
Anti-diabetic agents	0/27 (0%)	16/20 (80.0%)	
Insulin	–	5/16 (31.3%)	
Metformin	–	5/16 (31.3%)	
Sulfonylurea	–	1/16 (6.3%)	
Insulin and metformin	–	4/16 (25.0%)	
Metformin and sulfonylurea	–	1/16 (6.3%)	

*BMI* Body mass index, *HbAIC* Glycated hemoglobin, *MAP* Mean arterial pressure, *LVEDV* Left ventricular end-diastolic volume, *LVESV* Left ventricular end-systolic volume, *HR* Heart rate, *SV* Stroke volume, *CO* Cardiac output, *E* Early ventricular filling velocity, *A* Late ventricular filling velocity, *e’* Early ventricular myocardium relaxation velocity, *decT* Early ventricular deceleration time, *IVSd* Interventricular septal diameter diastole, *LVPWd* Left ventricular posterior wall thickness at diastole, *LVIDd* Left ventricular internal diameter at diastole, *LVIDs* Left ventricular internal diameter at systole. Data are presented as mean ± SEM. Unpaired T-test was performed. *p < 0.05; **p < 0.01; ***p < 0.0001 VS ND-CAD patients

**Fig. 1:**
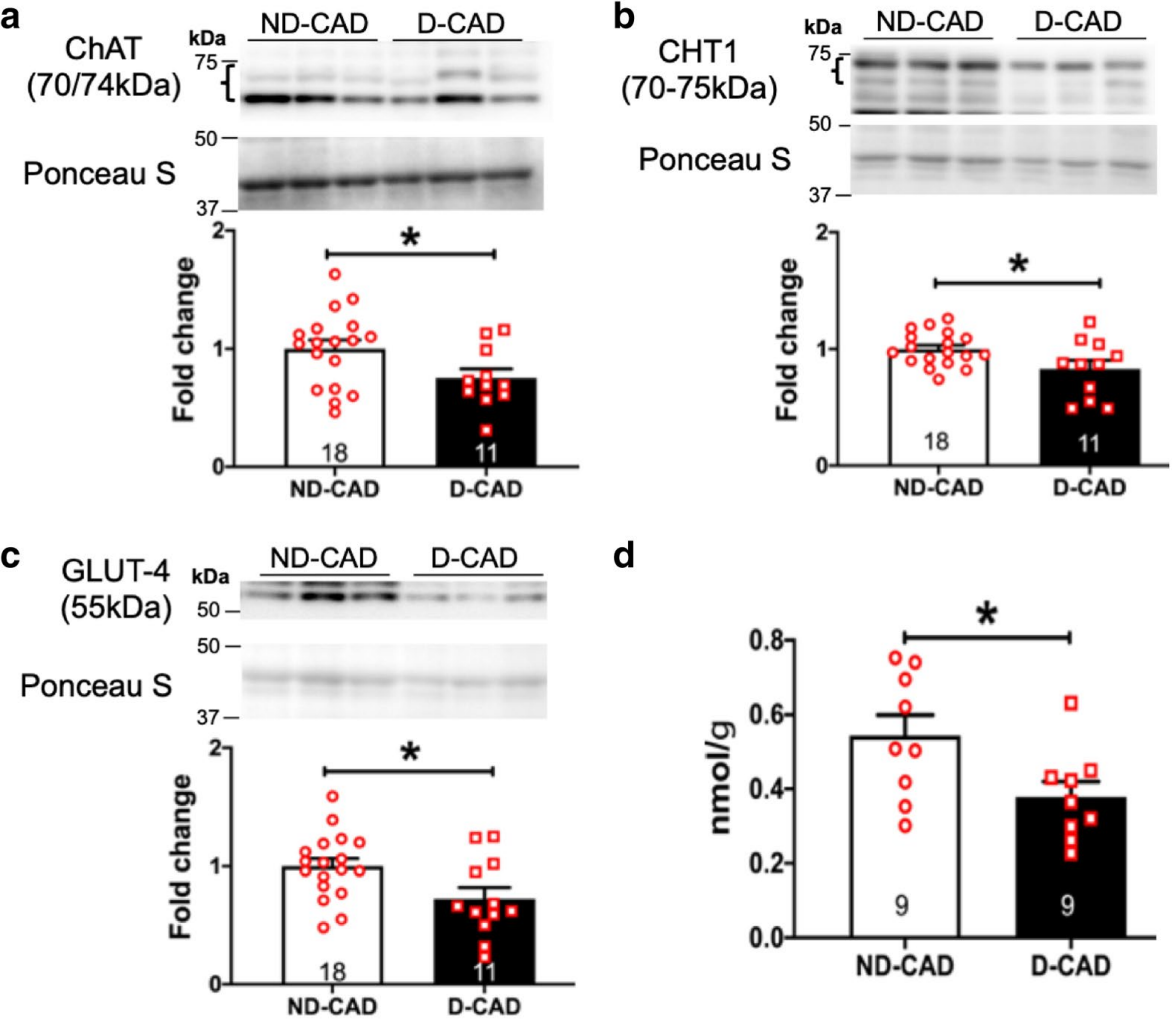
Cardiac NNCS and glucose content in the diabetic human ventricular tissues. Representative blots and quantitative bar graphs with scatter plots showing the protein expression of ChAT (**a**), CHT1 (**b**) and GLUT-4 (**c**) as well as the cardiac glucose content (**d**) in the ND- and D-CAD patients. Data are presented as mean ± SEM. Unpaired T-test was performed. *p < 0.05, **p < 0.01 vs ND-CAD patients. The number of samples per group is indicated in the figure

Immunoblotting experiments provided evidence for dysregulation of cardiac NNCS in the myocardium of diabetic patients. This is supported by significant reduction in the expression of ACh synthesizing enzyme ChAT (Fig. 1a and Additional file 1: Figure SIA) in D-CAD patients indicative of a reduced ability of the diabetic heart to synthesize ACh. Further, there was a significant decrease in the expression of CHT1 transporter in the diabetic human heart. CHT1 is responsible for the reuptake of choline, a rate-limiting step for ACh production (Fig. 1b and Additional file 1: Figure SIB). Importantly, these changes in expression of the LV of D-CAD patients were accompanied by a significant reduction in the expression of GLUT-4 (Fig. 1c and Additional file 1: Figure SIC) and lower cardiac glucose content (Fig. 1d).

There were no changes in the protein expression of VAChT, AChE and M_2_AChR in the D-CAD patients (Additional file 1: Figure SID–F). Nevertheless, the present data provide evidence for dysregulation of cardiac NNCS and impaired glucose homeostasis in the LV of D-CAD patients. A Pearson correlation analysis did not show any significant correlation between NNCS components and BMI, HbA1c, diabetes duration and EF. However, a positive correlation between CHT1 and BMI in the ND-CAD patients was observed (Additional file 1: Table S1).

**Fig. 2:**
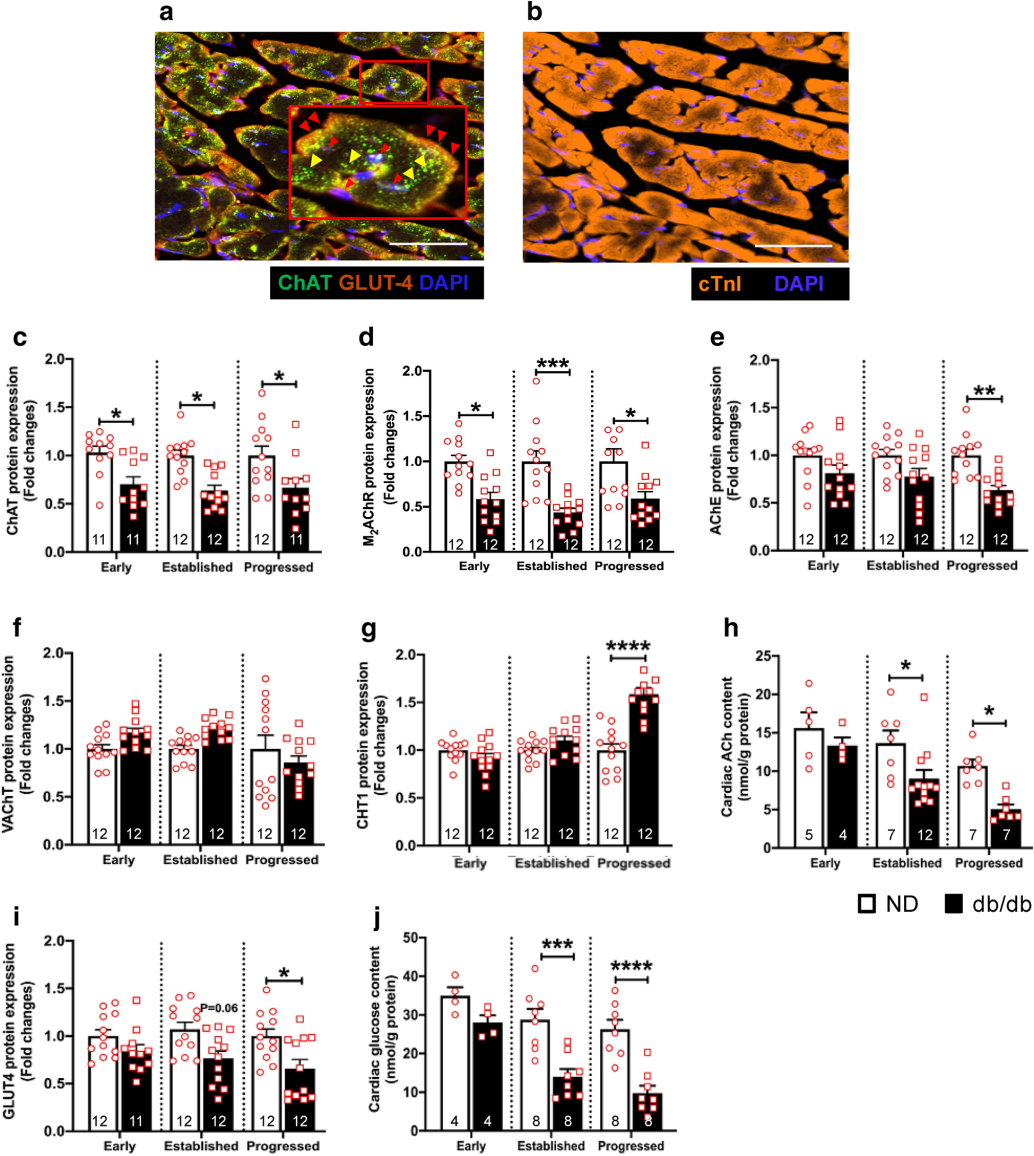
Dysregulation of cardiac NNCS in the diabetic db/db mouse heart. **a**, **b** Representative confocal images showing the localization of ChAT (yellow arrowhead) and GLUT-4 (red arrowhead) (**a**) as well as cardiac troponin I (cTnI, **b**) in the ND mouse ventricular tissues. Scale bars are 50 μm. **c–g**, **i** Quantitative bar graphs with scatter plots showing the protein expression of ChAT (**c**), M_2_AChR (**d**), AChE (**e**), VAChT (**f**), CHT1 (**g**) and GLUT-4 (**i**) in the ND and db/db mice at early, established and progressed stage of diabetes. Data are presented as mean ± SEM. Two-way ANOVA with Sidak’s test for multiple comparison was performed for all except GLUT-4, where Tukey’s test was performed. **h**, **j** Quantitative bar graphs with scatter plots showing the cardiac ACh content (**h**) and cardiac glucose content (**j**) in the LV of ND and db/db diabetic mice at different stages of diabetes. Two-way ANOVA with Sidak’s test for multiple comparison was performed. Data are expressed as mean ± SEM. Unpaired T-test was performed. *p < 0.05, **p < 0.01, ***p < 0.001 and ****p < 0.0001, vs age-matched ND mice. The numbers in the bars represent the number of samples and animals analyzed per group

Our next aim was to determine at which stage in the progression of diabetes, the expression of the components of cardiac NNCS and GLUT-4 are altered. For this, LV samples from type 2 diabetic db/db and age-matched ND mice were collected at three time points corresponding to early, established and progressed stage of diabetes. Using immunofluorescence analysis, we first identified the expression and localization of ChAT (yellow arrows) and GLUT-4 (red arrows) in the ND mouse LV tissue (Fig. 2a, b and Additional file 1: Figure SII). ChAT was localized in the cytoplasm of the cardiomyocytes, while GLUT-4 was localized in the perinuclear region and on the surface of cardiomyocytes. Next, the levels of expression of NNCS components (M_2_AChR, AChE, CHT1, ChAT, VAChT) and GLUT-4 were determined at the three stages of diabetes.

ChAT expression was significantly downregulated at all three time points, suggesting inability of the diabetic heart to sustain ACh synthesis (Fig. 2c and Additional file 1: Figure SIIIA). Interestingly, and in contrast to the human samples, the expression of M_2_AChR was consistently downregulated in db/db mice at all the time points (Fig. 2d and Additional file 1: Figure SIIIB). Considering the importance of the receptor for the initiation and transmission of cellular signaling pathways in response to ACh, this provides strong evidence for an impaired NNCS in the LV of db/db mice. With regard to other NNCS components, a downregulation of AChE (Fig. 2e and Additional file 1: Figure SIIIC) was observed in the progressed stage of diabetes. VAChT showed a downward trend in the progressed stage of diabetes, but this was not significant (Fig. 2f and Additional file 1: Figure SIIID). CHT1 was upregulated in the progressed stage of diabetes (Fig. 2g and Additional file 1: Figure SIIIE). Importantly, dysregulation of cardiac NNCS in the diabetic mouse heart was associated with reduced ACh content (Fig. 2h).

The protein expression of GLUT-4 was significantly decreased in the db/db mice heart in the progressed stage, suggesting reduced glucose uptake (Fig. 2i and Additional file 1: Figure SIIIF) that was further supported by reduced glucose content (Additional file 1: Figure SIIIJ).

In summary, in db/db mice, we observed a decreased ChAT and M_2_AChR expression from the early stage of diabetes, indicating reduced ACh level and ACh-mediated signaling. This was confirmed by a significant reduction of ACh content in the diabetic heart at the established and progressed stages (Fig. 2h). With the progression of diabetes, there was downregulation of other NNCS components VAChT and AChE, along with reduced GLUT-4 and cardiac glucose content.

Altogether, the current results from human and mouse hearts suggest dysregulation of cardiac NNCS by diabetes, suggesting its potential role in the development of diabetes-induced cardiovascular dysfunction. Therefore, our next aim was to determine how activation of NNCS in the diabetic heart effects cardiac and vascular function.

### Characterization of db/db-ChAT-tg mice

**Fig. 3:**
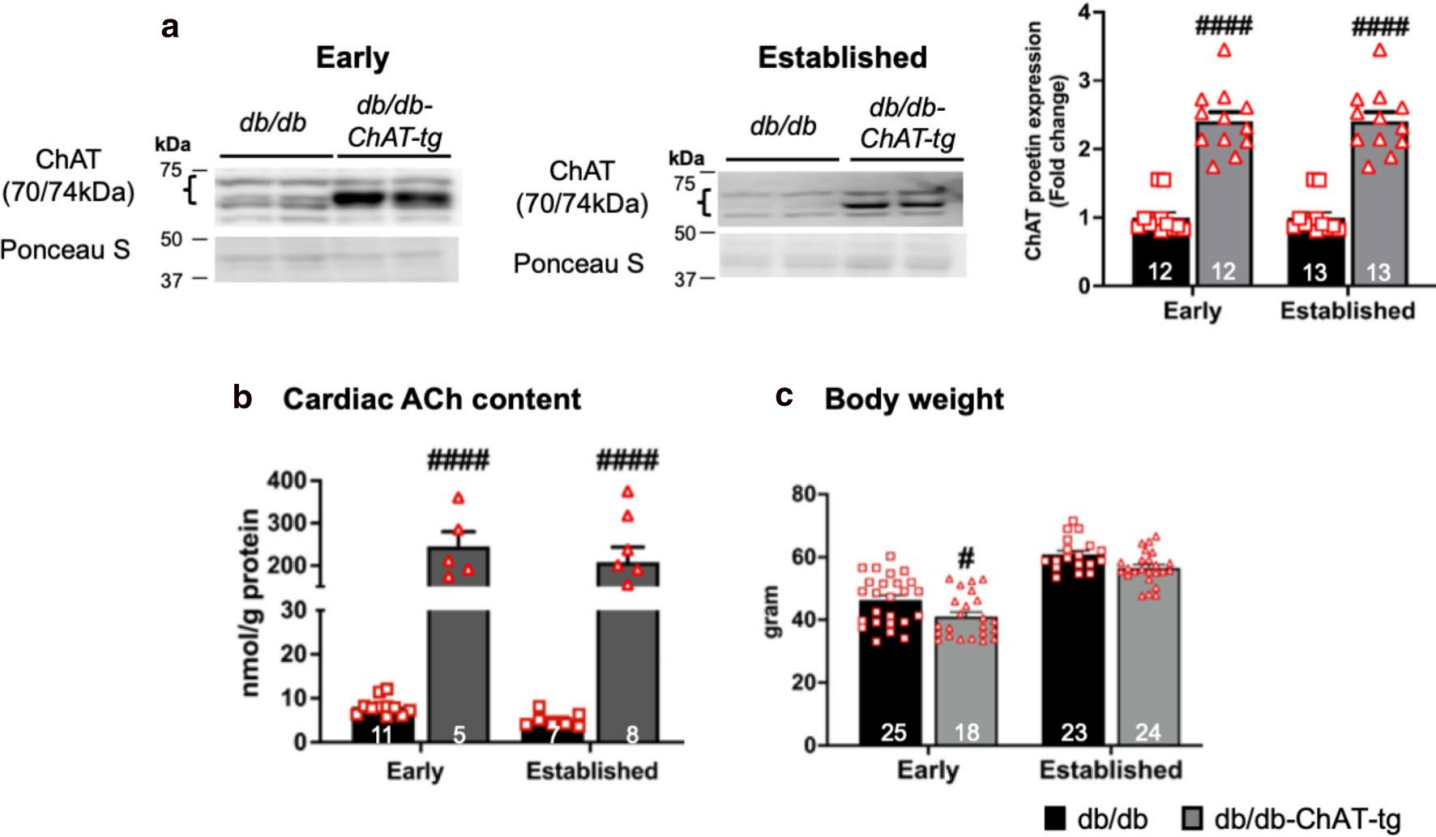
The characteristics of db/db-ChAT-tg mice. **a** Representative blots and quantitative bar graphs with scatter plots showing the protein expression of ChAT in the diabetic (db/db) and db/db-ChAT-tg heart at early and established stage of diabetes. A non-parametric Kruskal–Wallis test with Bunn’s test was performed. **b** The quantitative bar graphs with scatter plots showing the cardiac ACh content in the db/db and db/db-ChAT-tg mice at early and established stage of diabetes. Two way-ANOVA was performed. **c** Quantitative bar graphs with scatter plots showing the changes in body weight between the db/db and db/db-ChAT-tg mice at early and established stage of diabetes. Two-way ANOVA with Tukey’s test for multiple comparisons was performed. Data are expressed as mean ± SEM. #p < 0.05 and ####P < 0.0001 compared to age-matched db/db mice as indicated. The numbers in the bars represent the number of samples and animals analyzed per group

The db/db-ChAT-tg mice were generated as described in the methods section, to chronically activate the cardiac NNCS and hence increase the ACh level in the diabetic heart. We confirmed a twofold increase in protein expression of ChAT in the left ventricle of db/db-ChAT-tg mice (Fig. 3a, Additional file 1: Figures SIIA and SIVA). Importantly, this resulted in a ~ 17-fold increase in the ACh content in the db/db-ChAT-tg mice at two sampled time points (Fig. 3b). Although there was a significant reduction in the body weight at the early stage, there was no difference at the established stage of the disease compared with db/db (Fig. 3c).

Overall, these mice provide evidence for elevated cardiac ACh content, making them a suitable model to assess whether this has a positive impact on vascular and cardiac function in the diabetic heart.

### Activation of cardiac NNCS activated pro-survival and pro-angiogenic factors and prevented diabetes-induced downregulation of GLUT-4 in the heart

**Fig. 4:**
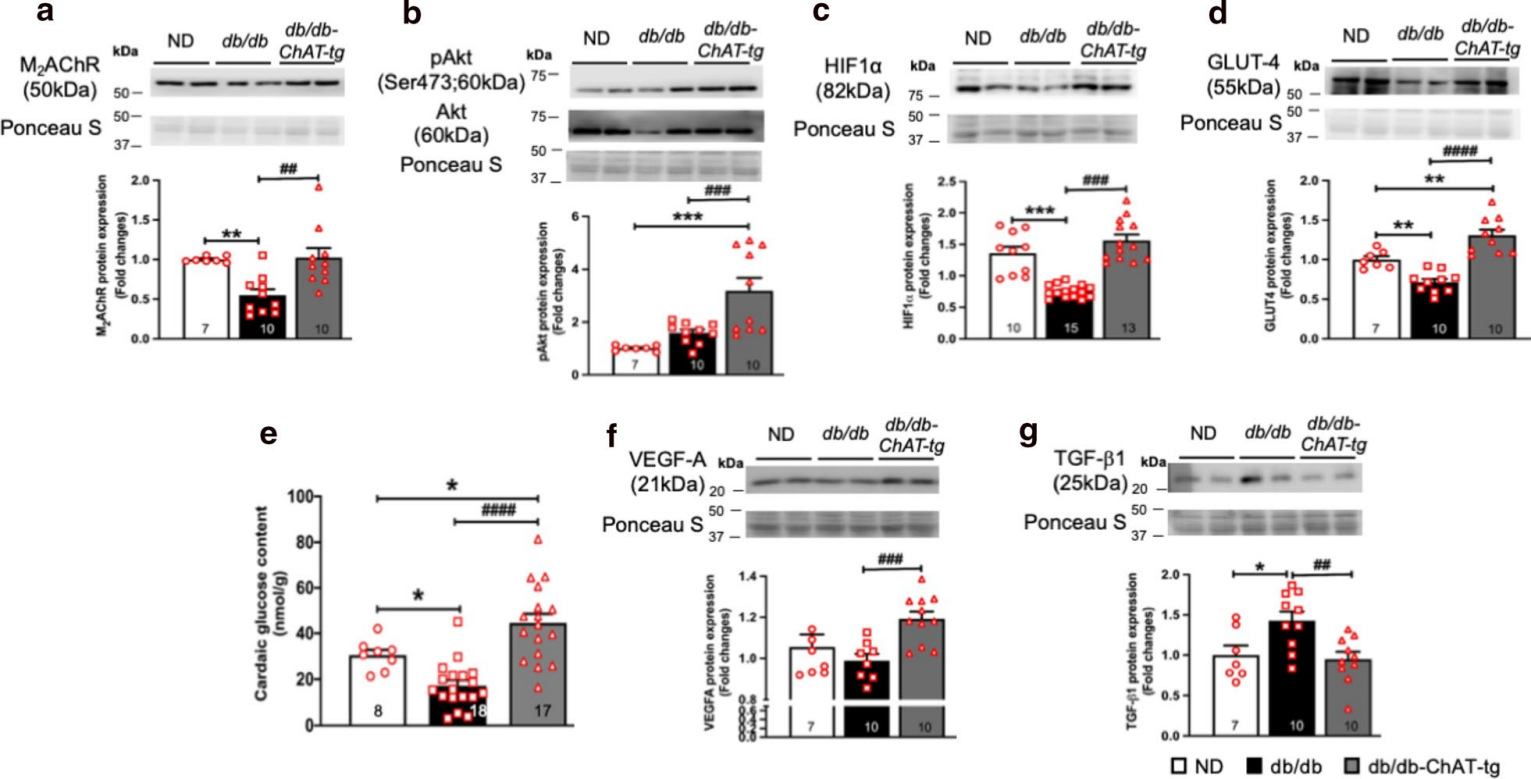
Activation of pro-survival and pro-angiogenic factors in the db/db-ChAT-tg mice. **a–d**, **f** Representative blots and quantitative bar graphs with scatter plots showing the protein expression of M_2_AChR (**a**), p-Akt and Akt (**b**), HIF-1α (**c**), GLUT-4 (**d**), VEGF-A (**f**) and TGF-β1 (**g**) in the ND, db/db and db/db-ChAT-tg mice. Data are presented as mean ± SEM. A non-parametric Kruskal–Wallis test with Bunn’s test was performed. **e** The cardiac glucose content was increased by 1.94-fold in the db/db-ChAT-tg mice in comparison to the db/db mice. Data are presented as mean ± SEM. One-way ANOVA with Tukey’s test for multiple comparisons was performed. *p < 0.05, **p < 0.01, ***p < 0.001 vs age-matched ND mice; ##p < 0.01,###p < 0.001; ####p < 0.0001 vs age-matched db/db mice; The number of samples per group is indicated in the figure

Activation of NNCS prevented the downregulation of M_2_AChR in the db/db-ChAT-tg heart at the established stage, showing a comparable expression to the ND mouse heart (Fig. 4a and Additional file 1: Figure SIVB). M_2_AChR activates the pro-survival Akt signalling cascade [28, 29]. In line with this, western blot analysis revealed increased Akt phosphorylation suggesting activated Akt (Fig. 4b and Additional file 1: Figure SIVC) and its downstream target HIF1α (Fig. 4c and Additional file 1: Figure SIVD) in db/db-ChAT-tg hearts. HIF1α forms a transcription factor with HIF1β that activates the transcription of GLUT-4 and VEGF to modulate glucose homeostasis [22] and angiogenesis [30], respectively. To establish whether this was the case, we analyzed GLUT-4 expression and cardiac glucose content. In contrast to db/db mice, GLUT-4 protein expression remained elevated in the db/db-ChAT-tg mice (Fig. 4d and Additional file 1: Figure SIVE). This was supported by preserved glucose content in db/db-ChAT-tg hearts (Fig. 4e). Additionally, pro-angiogenic VEGF-A protein expression was significantly increased in the db/db-ChAT-tg hearts in contrast to db/db heart (Fig. 4f and Additional file 1: Figure SIVF). Previous studies showed that ACh exerts anti-fibrotic effects via downregulation of pro-fibrotic transforming growth factor TGF-β1 [31, 32]. In our study, diabetes significantly increased TGF-β1 expression and this was significantly downregulated in db/db-ChAT-tg heart (Fig. 4g and Additional file 1: Figure SIVG).

Taken together, our data provide evidence that activation of cardiac NNCS prevented downregulation of M_2_AChR, activated Akt and HIF1α, as well as increased GLUT-4 and VEGF-A expression. Activation of these signaling molecules is indicative of improved cardiac metabolism and function. To reveal whether or not this translated into improved vascular and cardiac function vascular and cardiac function was further assessed.

### Activation of cardiac NNCS preserved coronary vasculature and vascular function in the diabetic heart

**Fig. 5:**
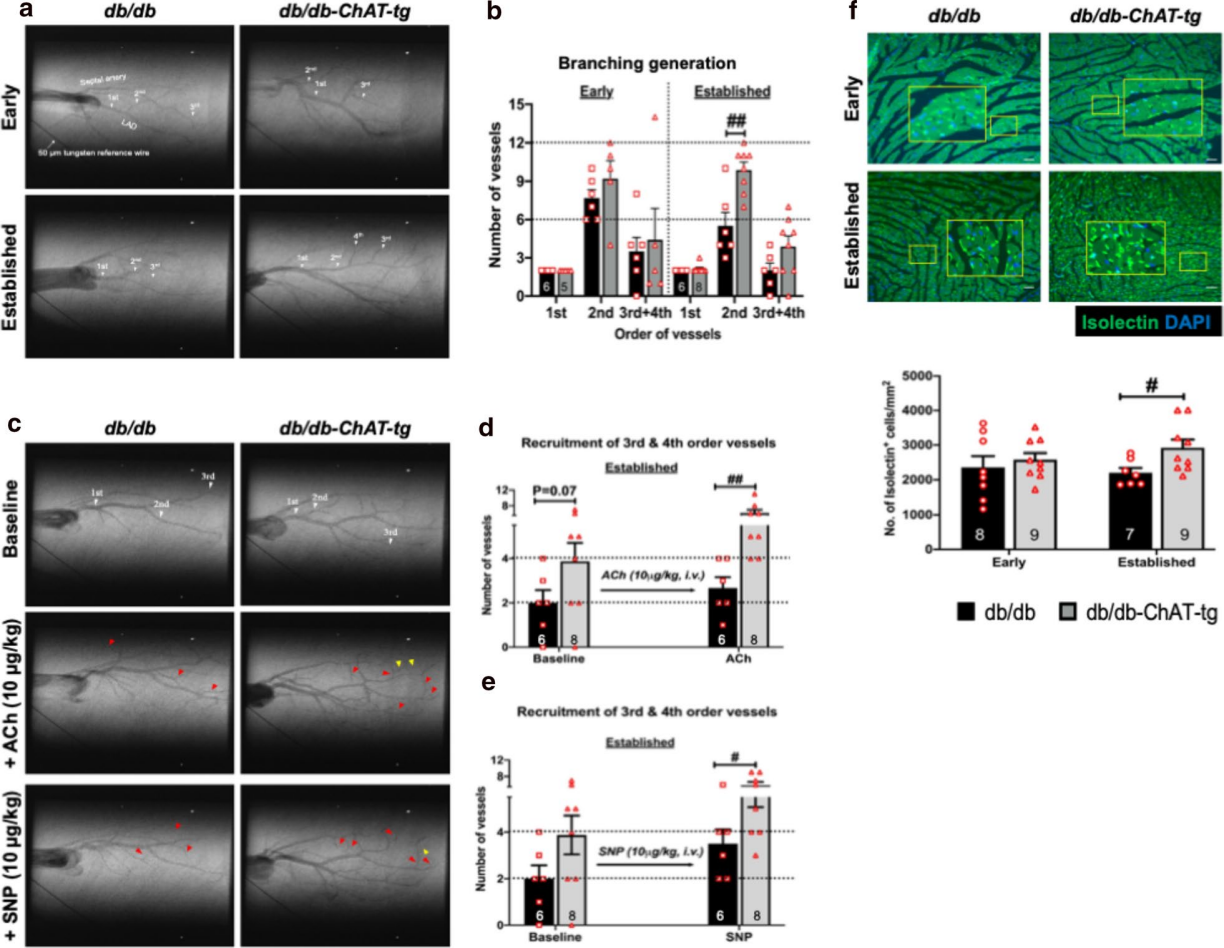
Preserved coronary vasculature in the db/db-ChAT-tg mice. **a**, **b** Representative microangiography images (**a**) and quantitative bar graphs with scatter plots showing the number (**b**) of first, second, third-order coronary vessels in the db/db and db/db-ChAT-tg mice at early and established stage of diabetes. The 50 μm diameter tungsten wire in the bottom left corner of all angiogram frames serves as a reference. **c** Representative microangiogram images showing the vasculature in the baseline condition, ACh infusion and SNP infusion in db/db and db/db-ChAT-tg mice at established stage of diabetes. Red arrows indicate third-order coronary vessels, while yellow arrows indicate fourth-order coronary vessels. **d**, **e** Quantitative bar graphs with scatter plots showing the number of third and fourth-order coronary vessels in the db/db and db/db-ChAT-tg mice (established stage) before and during ACh (**d**) and SNP (**e**) infusion. A non-parametric Mann–Whitney U test was performed. **f** Representative immunofluorescent images and bar graphs with scatter plots showing the density of capillaries indicated by the number of isolectin-stained endothelial cells in the myocardium of db/db and db/db-ChAT-tg mice at early and established stage of diabetes. Data are presented as mean ± SEM. #p < 0.05, ##p < 0.01 vs age-matched db/db mice. The number of samples per group is included in the bars

We examined the number of coronary vessels and their responsiveness when exposed to vasodilators in db/db-ChAT-tg mice *in-vivo* using Synchrotron radiation microangiography. While there was no difference in the total number of coronary vessels between db/db-ChAT-tg and db/db mice in the early stage of diabetes (Fig. 5a, b), however more second-order coronary vessels were observed in db/db-ChAT-tg mice with established diabetes (Fig. 5a, b).

To assess vessel responsiveness, the change in number of angiographically visualized third and fourth-order coronary vessels was determined during sequential acute infusions of ACh (Fig. 5c, d) and SNP infusion (Fig. 5c, e). The number of visible vessels at baseline was twice as high in db/db-ChAT-tg compared to the age-matched db/db heart (p = 0.07, Fig. 5c–e). Further, in db/db animals the infusion of ACh or SNP had only a small effect in recruiting new vessels, whereas in the db/db-ChAT-tg there was at least a 1.5-fold increase in the number of visible vessels counted (Fig. 5c–e). This suggests enhanced vascular responsiveness due to preserved endothelial and smooth muscle cell function in small coronary arteries in db/db-ChAT-tg heart. Vascular responsiveness of larger coronary vessels was unchanged compared with db/db animals (Additional file 1: Figure SVB–E).

In addition to improvements in arterial endothelial function, *ex-vivo* capillary density in hearts of db/db-ChAT-tg mice were compared to those of the db/db mice. No differences were observed at early stage of diabetes, whereas the capillary density was significantly higher in db/db-ChAT-tg mouse LV at the established stage of diabetes (Fig. 5f). The arteriole density remained unchanged (Additional file 1: Figure SVI).

### Reduced cardiac fibrosis in db/db-ChAT-tg mice

**Fig. 6:**
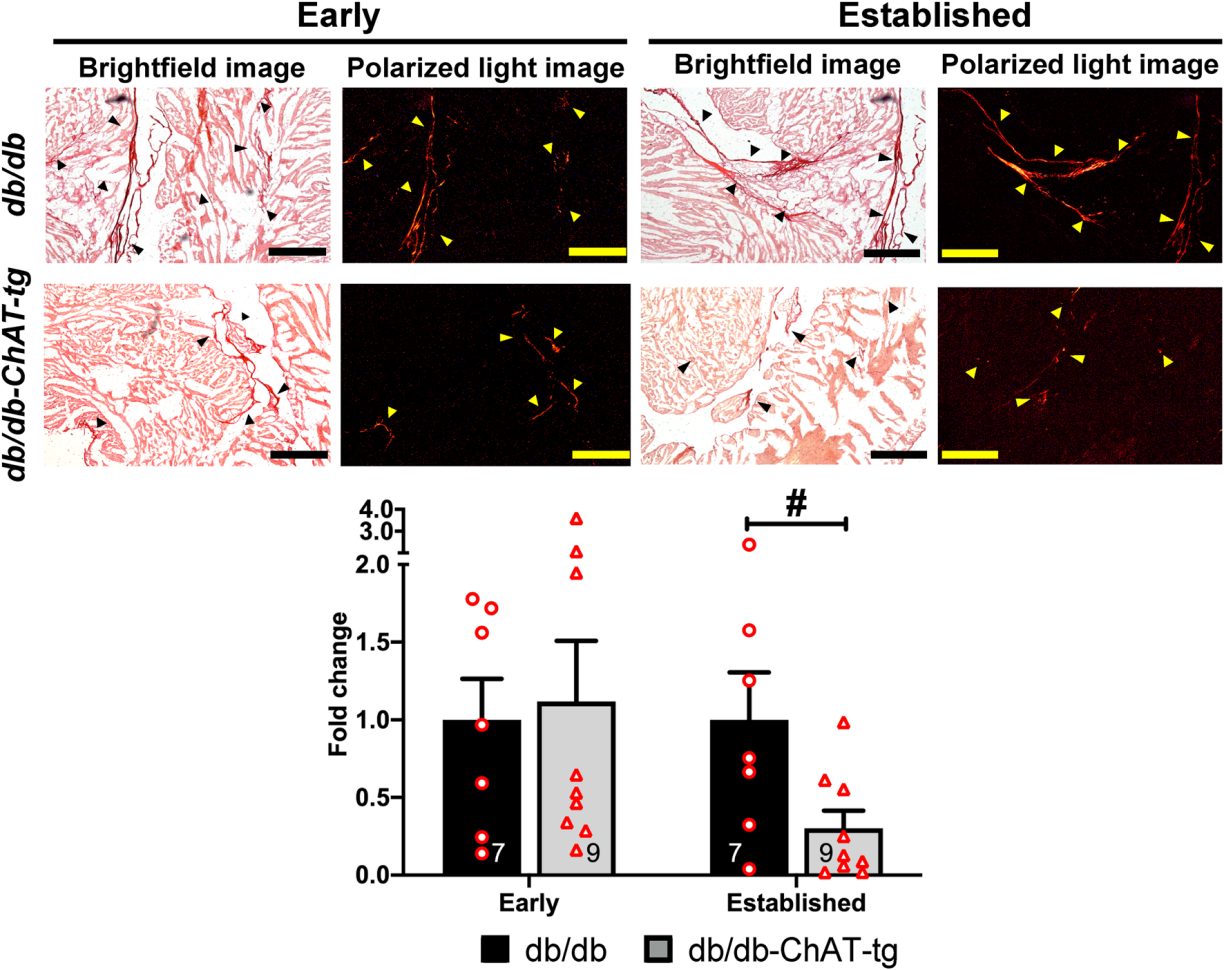
Reduced fibrosis in the db/db-ChAT-tg mice heart. Representative brightfield and polarized images from picrosirius staining showing the cardiac fibrosis in the db/db and db/db-ChAT-tg mice at early and established stage of diabetes. The bar graphs with scatter plots summarize the fold change of fibrotic area in the db/db and db/db-ChAT-tg mice hearts at both stages. Data are presented as mean ± SEM. A non-parametric Mann–Whitney U test was performed. #p < 0.05 vs aged-matched db/db mice. The number of samples per group is indicated in the figure

Diabetes leads to progressive loss of cardiomyocytes, which are replaced by fibrotic scar tissue in the heart impairing its contractility [33, 34]. Our group previously reported increased cardiac fibrosis from 20-weeks of age in db/db mice relative to ND mice [1]. While there was no difference in the level of fibrosis in the early stage of diabetes, the downregulation of TGF-β1 in the established stage of the db/db-ChAT-tg heart translated to a significant reduction in cardiac fibrosis at the established stage of diabetes (Fig. 6).

### Activation of cardiac NNCS enhanced the cardiac function in the diabetic heart

**Fig. 7:**
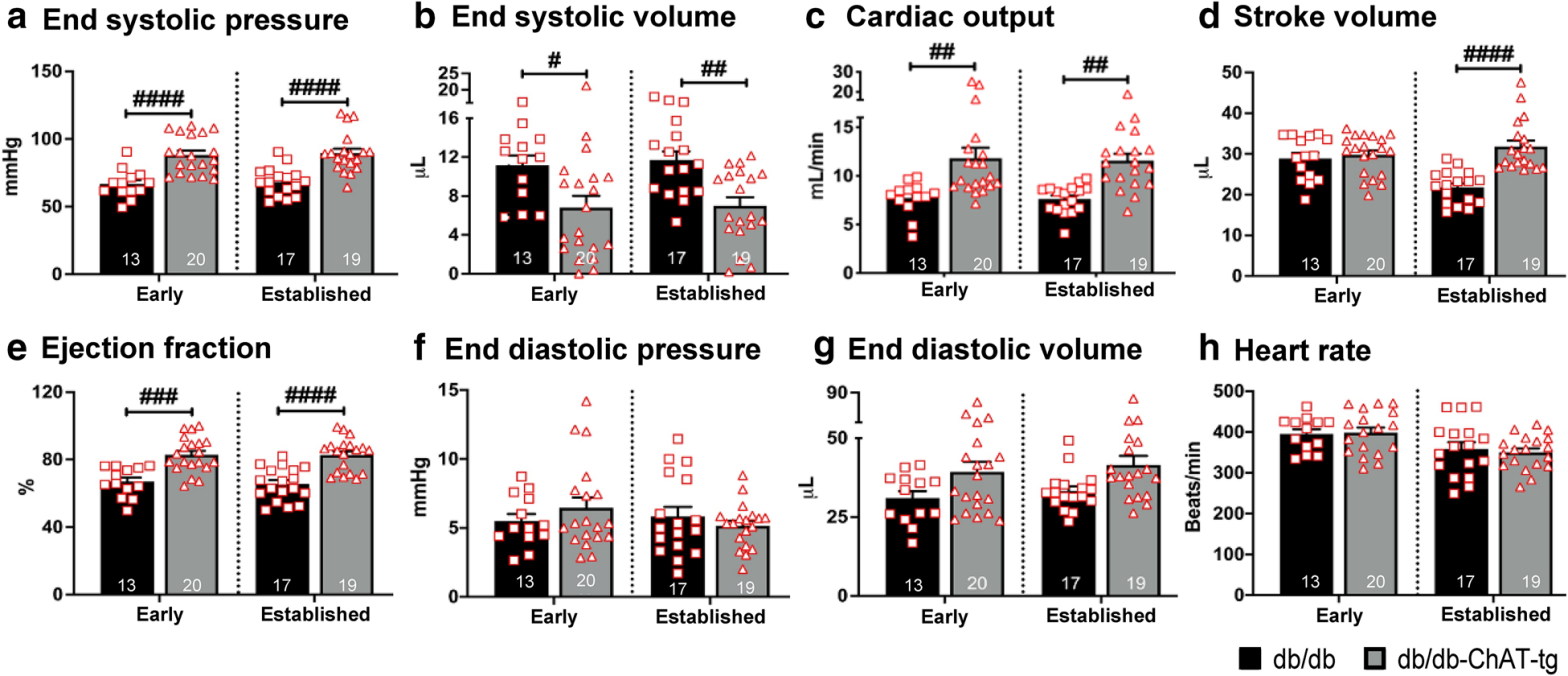
Improved cardiac function of db/db-ChAT-tg mice. Bar graphs with scatter plots showing end-systolic pressure (**a**), end-systolic volume (**b**), cardiac output (**c**), stroke volume (**d**), ejection fraction (**e**), end-diastolic pressure (**f**), end-diastolic volume (**g**) and heart rate (**h**) in the db/db and db/db-ChAT-tg mice at early and established stage of diabetes. Data are expressed as mean ± SEM. Two-way ANOVA with Sidak’s test for multiple comparisons was performed. #p < 0.05; ##p < 0.01; ###p < 0.001; ####p < 0.0001 vs age-matched db/db mice. The number of samples per group is indicated in figure

Finally, we determined if the identified beneficial effects observed so far translate into improved cardiac function. PV loop analysis showed a significant increase in the end-systolic pressure (ESP) (Fig. 7a) and a considerable decrease in the end-systolic volume (ESV) in the db/db-ChAT-tg mice (Fig. 7b). As a result, the cardiac output (CO), stroke volume (SV) and ejection fraction (EF) were considerably improved in the db/db-ChAT-tg mice (Fig. 7c–e). There was no difference in the indices of diastolic function, such as end-diastolic pressure (EDP) and volume (EDV) (Fig. 7f, g) and heart rate (Fig. 7h). Taken together, these results suggest that activation of cardiac NNCS in the db/db-ChAT-tg mice preserved LV systolic function and contractility in comparison to the age-matched db/db mice, possibly through activation of pro-survival signaling cascade, improved angiogenesis, increased cardiac glucose content and reduced fibrosis as a result of increased ACh content through overexpression of the *ChAT* gene.

## Discussion

To the best of our knowledge, this is the first study to characterize the changes in the components of cardiac NNCS in T2DM. Our results provide unique evidence for alteration of the cardiac NNCS in the human and mouse diabetic heart. We also showed that cardiac-specific overexpression of the *ChAT* gene increased cardiac ACh content and induced upregulation of Akt/HIF1α signaling and preserved GLUT-4 and VEGF-A expression. As a consequence, db/db-ChAT-tg mice exhibited preserved coronary macro- and microvasculature, reduced cardiac fibrosis and increased cardiac glucose content, overall resulting in improved cardiovascular function in the diabetic heart.

The cardiac NNCS comprises of different components to maintain ACh homeostasis and transduction of signaling in the cardiomyocytes [13–15]. Previous studies showed that cardiomyocytes utilize ACh as an auto-/paracrine mediator to regulate glucose metabolism through muscarinic ACh receptors in the heart [17, 18]. In our study, diabetes-induced dysregulation of different components of cardiac NNCS and hence reduced myocardial ACh content at both established and progressed stages of T2DM was observed. Interestingly, we observed differences in the components of cardiac NNCS that were altered in the human (i.e. CHT1) and mouse (i.e. AChE and M_2_AChR) hearts as a consequence of T2DM. While this discrepancy could be species-specific or a result of associated ischemia or drug treatment in the humans with existing cardiovascular disease, the common consequences of these changes in both species indicate a reduction in cardiac ACh content in the diabetic heart. The diabetic mice exhibited both diastolic and systolic dysfunction in later stages as previously reported [3], and we observed similar diastolic and systolic dysfunction in the cohort of diabetic patients reported in our study (Table 1). T2DM patients whose cardiac samples were used in this study were mainly prescribed with either metformin or insulin. While the exact interaction between these drugs and ACh metabolism is not clear, we observed an increase in ChAT expression in response to insulin stimulation, in cultured human cardiomyocytes, which was not observed in cardiomyocytes exposed to conditions that mimicked diabetes (Additional file 1: Figure SVIIB). Similarly, previous studies have demonstrated that metformin can inhibit the activity of AChE in human erythrocytes and suppress sympathetic activity [35], although diabetic individuals often develop cardiac autonomic neuropathy. Therefore, while it is plausible that anti-diabetic drugs may have a positive interaction with ACh metabolism, this is likely to be impaired under diabetic condition, although further studies are required to demonstrate this direct link. While the underlying mechanism(s) that cause NNCS dysregulation in T2DM is/are not clear, preliminary evidence suggests that excessive intracellular buildup of lipid and its intermediates such as ceramide and diglyceride might possibly disrupt signaling cascade that regulate NNCS in the cardiomyocytes (Additional file 1: Figure SVIIB) [36].

In T2DM, impairment in insulin receptor substrate (IRS)-1/PI3K/Akt signaling cascade decreases insulin-stimulated GLUT-4 translocation hence reducing the glucose uptake and metabolism in cardiomyocytes [7]. Previous studies showed that overexpression of *GLUT-4* gene enhanced glycolysis and glucose oxidation as well as suppressed palmitate oxidation, thereby preventing the onset of diabetes-induced cardiac dysfunction [37–39]. In our study, activation of cardiac NNCS via cardiac-specific overexpression of *ChAT* gene in the diabetic heart resulted in elevated GLUT-4 expression and cardiac glucose content. This is in agreement with previous studies showing that cardiac NNCS upregulates GLUT-4 expression in cellular and murine models following overexpression of *ChAT* gene [13, 17, 18], or, downregulation of GLUT-4 expression and impaired glucose metabolism in cardiac-specific ChAT knockdown mice [23]. Additionally, our present study further provides evidence for ACh mediated activation of pAkt and HIF1α expression in db/db ChAT-tg mice to regulate GLUT-4 expression and hence increase glucose uptake [18, 19, 23]. Glucose uptake is compromised in diabetic individuals due to the slow rate of glucose transport across the membrane to the cytosol, secondary to the reduction in myocardial GLUT4. While we did not measure the rate of glucose transport *in-vivo*, human cardiomyocytes cultured under diabetic conditions showed a marked reduction in membrane GLUT4 expression in response to insulin stimulation, suggesting reduced glucose uptake in these cells. However, overexpression of human *ChAT* gene restored membrane GLUT4 expression (Additional file 1: Figure SVII). Furthermore, the PI3K/Akt signaling cascade plays a crucial role in maintaining cardiac function. Previous studies revealed that cardiomyocytes from mice overexpressing Akt exhibited increased contractility [40] and pharmacological activation of PI3K/Akt signaling cascade ameliorated diastolic dysfunction [41]. Thus, in addition to enhanced GLUT-4 expression and glucose content in db/db-ChAT-tg heart, increased pAkt expression may also regulate other signaling cascades that are beneficial to cardiac function.

Diabetes is also associated with coronary macrovascular [10] and microvascular rarefaction [9], which, together with impaired contractility drives the development of DHD [10]. In the current study, activation of cardiac NNCS prevented the reduction in coronary vessels in the heart at the later stage of diabetes, which is likely through the activation of PI3K/Akt/ HIF1α signaling cascade and its downstream target pro-angiogenic VEGF as previously demonstrated [18, 20, 30]. This is in agreement with our previous study demonstrating that reduced VEGF-A protein expression preceded the reduction in capillaries and small arterioles in the db/db mouse heart [2]. Another interesting observation was a trend of increasing diameter of coronary vessels in the db/db-ChAT-tg mice at baseline condition (Additional file 1: Figure SVA). While the exact reason for this is not known, our previous study showed an increased level of nitric oxide (NO), which is also a potent vasodilator [42, 43], in the ChAT-tg mice [25]. In the present study, overexpression of the *ChAT* gene induced at least a 17-fold increase in ACh content in the heart. Although the NO level was not measured, it is known that NO is a result of ACh-mediated muscarinic receptor stimulation [15, 20, 44]. Considering that coronary endothelial cells express nicotinic and muscarinic ACh receptors [45], it is likely that elevated ACh increases the release of NO from endothelial cells in the db/db-ChAT-tg heart, contributing to the increased diameter of coronary vessels [46].

Cardiac fibrosis is a pathological condition caused by excessive accumulation of extracellular matrix (ECM) components [47]. This results in myocardial stiffening, affecting cardiac contractility. Extensive evidence has demonstrated the major role for myocardial fibrosis in inducing contractile dysfunction in individuals with diabetes [48]. Previous study showed that anti-ischemic agent trimetazidine, which improves myocardial glucose utilization, reduced cardiac apoptosis and fibrosis in the rat model of T2DM [49]. ACh has been shown to exert anti-fibrotic effects via downregulation of pro-fibrotic transforming growth factor TGF-β1 [31, 32]. TGF-β1 induces signaling cascade to activate transcription factor Smad2 and Smad3, thus upregulating downstream genes that are involved in myofibroblast differentiation [50, 51]. Studies have demonstrated the crucial role for TGF-β1 induced fibrosis in the development of diabetic cardiomyopathy [52]. In our study, the fibrotic proportional area was significantly decreased in the db/db-ChAT-tg heart, which could be partially due to enhanced energy preservation via glucose metabolism and superior myocardial perfusion, eventually preventing the progressive loss of cardiomyocytes and partly through the direct effect of ACh in downregulating the expression of TGF-β1. Moreover, cardiac fibroblasts express α7 nicotinic ACh receptor, suggesting that ACh could directly suppress cardiac fibroblasts from differentiating into myofibroblasts [53].

Another observation from our study was the reduction of body weight in the db/db-ChAT-tg mice in the early stage of diabetes, indicating that elevated ACh from the heart has other extra-cardiac effects as well. This is supported by evidence from Oikawa et al. [25, 54] reporting activation of the central nervous system via the afferent vagus nerve in ND ChAT-tg mice. They showed increased c-Fos signal in the medulla and increased norepinephrine (NE) level in the hypothalamus [25] in these animals. Hypothalamic NE plays a role in suppressing food intake [55]. Therefore, it may be hypothesized that weight loss observed in the db/db-ChAT-tg mice could be due to suppressed food intake as a result of increased hypothalamic NE as a consequence of increased cardiac ACh. Additionally, reduced body weight in db/db-ChAT-tg mice at the early stage of diabetes may have partially contributed to improved cardiac function as well as altering the molecular mechanisms. We did not further investigate this as it is beyond the scope of our present study. However, considering that the cardiac function of db/db-ChAT-tg mice remained improved despite the body weight was similar to db/db mice at the later stage of diabetes, this suggests that changes in body weight may not play a major role in influencing the cardiac function of db/db-ChAT-tg mice.

Our study has a few limitations. First, we did not include ND db/ + mice as a control group to compare the cardiovascular function with db/db mice and db/db-ChAT-tg mice. However, we have previously reported a progressive deterioration of cardiac [3, 56, 57] and coronary function [10] in these db/db mice in comparison to ND mice. Second, our study demonstrated the pleiotropic effect of ACh in the db/db-ChAT-tg mouse heart. While the experimental design of our current study did not allow us to specifically distinguish the cell type(s) responsible for the beneficial effects, this might be something to explore in future studies. Third, the present study used db/db-ChAT-tg mice as a model to activate cardiac NNCS to examine its role in the diabetic heart. Future study with the use of atropine (i.e. muscarinic ACh receptor antagonist) or cardiac-specific knockout of *ChAT* gene can provide complimentary information whether antagonizing ACh-mediated signaling cascade prevents the positive effects seen in the db/db-ChAT-tg mice.

## Conclusion

Our study shows that in diabetes alterations of cardiac NNCS occur. These alterations seem to contribute to impaired Akt and HIF1α signaling, leading to impaired contractility and vascular function and likely contribute to the development of DHD. We provide evidence that activation of cardiac NNCS via overexpression of the *ChAT* gene and increased cardiac ACh is beneficial in the diabetic heart. The beneficial effects include activation of pro-survival signaling, preservation of vasculature structure and function, through an increase of HIF1α and VEGF-A and increased cardiac glucose content by increasing GLUT-4 expression. These effects eventually prevented the progressive loss of cardiomyocytes, thereby reducing fibrosis and preserving cardiac function (Summarized in Additional file 1: Figure SX). These results altogether provide a strong foundation showing that targeting cardiac NNCS to increase cardiac ACh content could be a potential therapeutic approach to protect the diabetic heart.

## Supplementary Information

**Additional file 1.** Expanded methods and additional figures.

## Data Availability

The datasets used and/or analysed during the current study are available from the corresponding author on reasonable request.

## Abbreviations

Ach: Acetylcholine
AChE: Acetylcholinesterase
Akt: Protein kinase B
ATP: Adenosine triphosphate
CABG: Coronary artery bypass-grafting
CAD: Coronary artery disease
ChAT: Choline acetyltransferase
CHT1: Choline transporter1
CMVD: Coronary microvascular disease
CO: Cardiac output
CTNI: Cardiac troponin I
DAPI: 4′, 6′-Diamidino-2-phenylindole
db/db: C57BL/KsJ-Lepr^db/db^ mouse
db/db-ChAT-tg: C57BL/KsJ-Lepr^db/db^ –ChAT-tg mouse
D-CAD: Type-2 diabetic patient with coronary artery disease
DHD: Diabetic heart disease
EF: Ejection fraction
End-diastolic pressure: EDP
End-diastolic volume: EDV
End-systolic pressure: ESP
End-systolic volume: ESV
FFA: Free fatty acid
GLUT-4: Glucose transporter-4
HIF: Hypoxia-inducible factor
HIF1α: Hypoxia-inducible factor 1 α-subunit
HPLC: High-performance liquid chromatography
HR: Heart rate
LV: Left ventricle
M_2_AChR: Type-2 muscarinic acetylcholine receptor
ND: Non-diabetic mouse
ND-CAD: Non-diabetic patient with coronary artery disease
NE: Norepinephrine
NNCS: Non-neuronal cholinergic system
NO: Nitric oxide
PI3K: Phosphatidylinositol-3-kinase
PV: Pressure volume
SEM: Standard error of the mean
SNP: Sodium nitroprusside
SV: Stroke volume
T2DM: Type-2 diabetes mellitus
VAChT: Vesicular acetylcholine transporter
VEGF-A: Vascular endothelial growth factor-A
α-SMA: Alpha-smooth muscle actin
