# Supplementary material for: Activation of the cardiac non-neuronal cholinergic system prevents the development of diabetes-associated cardiovascular complications
Source: Cardiovasc Diabetol. 2021 Feb 22;20:50. doi: 10.1186/s12933-021-01231-8 (PMC7898760; doi:10.1186/s12933-021-01231-8)

Additional File 1

Expanded Materials and Methods

Ethics

The Animal Ethics Committee from the University of Otago (AEC25/12) and Nippon Medical School (27-003) approved the use of animals in this study. Health and Disability Ethics Committee in New Zealand approved the use of human left ventricular (LV) tissues (LRS/12/01/001/AM13). The human LV tissues of patients with or without type-2 diabetes with coronary artery disease (CAD) who underwent coronary artery bypass grafting (CABG) surgery in Dunedin hospital were collected following their informed consent.

Type-2 diabetic human ventricular tissues

The human ventricular tissues were collected from type-2 diabetic and non-diabetic patients with CAD undergoing on-pump coronary artery bypass graft surgery at Dunedin Hospital. The selection criteria of diabetic patients were hemoglobin A1C (HbA1C) level ≥ 50nmol/mol and diabetic duration of more than one year[1].

Animal models

As described in our previous studies[2, 3], diabetic C57BL/ksJ-lepr+/+ (db/db) and their non-diabetic C57BL/ksJ-lepr + littermates (db/+) (Jackson Laboratory) were used as the model of type-2 diabetes and age-matched controls, respectively[2, 3]. The ventricular tissues of db/db and non-diabetic (ND) db/+ mice at early (12-16 weeks), established (20-24 weeks) and progressed (28-32 weeks) of diabetes were used for western blot analysis.

The mouse with cardiac-specific overexpression of murine *ChAT* gene (ChAT-tg) was previously reported as the model of activated cardiac NNCS[4]. These ChAT-tg mice were crossbred with the heterozygous db/+ mice to generate heterozygous db/+ mice with ChAT transgene. These heterozygous littermates were further crossbred to generate homozygous db/db mice with cardiac-specific ChAT transgene (db/db-ChAT-tg). This unique model was maintained in the Department of Bioregulatory Science, Nippon Medical School and used as the model of type-2 diabetes with activated cardiac NNCS. After measurement of body weight, blood glucose level and LV function, the ventricular tissues of db/db-ChAT-tg mice and control diabetic db/db mice at early and established stage of diabetes were collected for western blot analysis. The wild type non-diabetic (ND) mice were included for western blot analysis only to allow comparison with db/db and db/db-ChAT-tg mice. Also, the Synchrotron radiation microangiography was performed on db/db and db/db-ChAT-tg mice at early and established stage of diabetes in the SPring-8 facility in Japan. The ventricular tissues from these mice were then retrieved and used to examine the density of arterioles and capillaries, cardiac fibrosis, cardiac glucose level and western blot analysis (i.e., VEGF-A).

Measurement of cardiac ACh level

The cardiac ACh level was detected by high-performance liquid chromatography (HPLC) as previously described[4]. Briefly, the left ventricle was harvested and homogenized in 0.1 M perchloric acid containing 0.1 mM AChE inhibitor physostigmine and internal control of isopropylhomocholine ($5x10^{-7}$ M). The tissue lysate was centrifuged and followed by filtration using a 10K centrifugal filter column (Amicon Ultra, UFC501096). Then, ten μL of the filtered lysate was injected into an HPLC system (HTEC-500; Eicom, Japan). The results were expressed as molarity (M).

Measurement of cardiac glucose level

The cardiac glucose level was measured using a glucose assay kit (Abcam, ab65333). According to the manufacturer's protocol, 50-100 mg of left ventricular tissues from ND-CAD and D-CAD patients as well as db/db-ChAT-tg and db/db mice (early and established stage of diabetes) were homogenized in 100 μL of assay buffer. The tissue lysates were centrifuged, deproteinized by 1 M perchloric acid and subsequently neutralized by ice-cold 2 M potassium hydroxide. To prepare a standard curve for the assay, 1 nmol/μL of glucose standard solution was prepared freshly and followed by serial dilution of this solution. Then, 50 μL of standards or test samples were added to wells containing 50 μL of reaction mix in a 96-well plate (BioRad, 5000205), and followed by 30 minutes of incubation at 37°C protected from light. The absorbance of glucose standards and test samples were measured at 570 nm by SpectraMax i3x microplate reader (Molecular Device, USA). A standard graph was plotted by using the absorbance of glucose standards versus the final concentration, thus allowing determination of the glucose concentration of test samples. The results were expressed as nmol/g.

Evaluation of cardiac function using pressure-volume (PV) catheter

The cardiac function was measured as described by Oikawa et al.[5]. Briefly, the db/db-ChAT-tg and db/db mice (early and established stage of diabetes) were anesthetized with isoflurane (1-2%) at a flow rate of 0.5-1.0 L/min. The incision was made into the abdominal cavity to allow the PV catheter to be inserted into the apex of the heart via the diaphragm. The catheter was connected to an ADVantage PV system control box (Transonic Systems) and a data acquisition system (iWorx system). Hemodynamic parameters were measured using LabScribe2 software (iWorx system).

Synchrotron radiation coronary microangiography

This experiment was performed to visualize and measure the functions of the *in-vivo* coronary circulation. The experiment was performed at the SPring-8 facility, BL28B2 beamline, Hyogo, Japan, using established protocols from our previous study[6]. The study was blinded by generating a unique number to each mouse to avoid bias on sample type during the analysis.

The db/db-ChAT-tg and db/db mice (early and established stage of diabetes) were anesthetized with isoflurane (1.5 – 5%) at a flow rate of 1L/min and positioned on a heating pad. The trachea was cannulated to mechanically ventilate the mouse. The jugular vein was cannulated with PE20 catheter for administration of fluid or drugs. The right common carotid artery was cannulated with a fine PE10 catheter for injection of iodinated contrast medium (Iomeron 350, Bracco-Eisai Co., Ltd.). Following surgical preparation, the mouse was positioned supine in front of, and perpendicular to the SATICON X-ray detector (Hitachi Denshi Techno-system) to image the thorax in an alignment of 9.5 mm x 9.5 mm imaging field [6]. A 50 μm-thick tungsten filament was placed across the corner of the detector’s window to be captured in all images and used as a reference to determine the size of the coronary vessels.

A baseline angiogram of the coronary circulation was first recorded before the infusion of drugs. Then, the mouse was infused with the endothelium-dependent vasodilator ACh (10 μg/kg/min) for five minutes, followed by injecting the contrast medium to record the coronary circulation. Next, the mouse was infused with sodium nitroprusside (SNP, 10 μg/kg/min), an endothelium-independent vasodilator, for five minutes, and the same procedure was repeated to record the coronary circulation.

Two analyses were performed using ImageJ software. First, the number of coronary vessel branches were counted. Second, the diameter of 2-4 vessels of each branch generation (i.e., first, second, and third-order vessels) were measured. The 50 μm-thick tungsten filament was used as a reference to measure the diameter of vessels. Finally, vessels were further sorted according to their size (0-100, 100-150, 150-200, and 200-350 μm). All the data are expressed as mean ± standard error of the mean, except the diameter of coronary vessels were expressed as the median in box and whisker to present the minimum and maximum distribution of data.

Western blot analysis

Mouse and human ventricular samples were homogenized in ice-cold RIPA buffer followed by centrifugation to obtain clear protein lysates. The lysates were resolved by SDS-PAGE and transferred onto polyvinylidene fluoride (PVDF) membrane (BioRad, 1620264). The total protein on the PVDF membranes was stained with Ponceau S solution and captured using a Syngene Pxi imaging system (USA). The membranes were probed with following primary antibodies overnight: goat anti-ChAT (Chemicon, AB144P; 1:1,000), rabbit anti-M_2AChR (Abcam, AB109226; 1:2,000), rabbit anti-AChE (Bioss, BS2511R; 1:1,000), rabbit anti-CHT1 (Merck Millipore, ABN458; 1:1,000), rabbit anti-GLUT-4 (NovusBio, NBP149533; 1:2,000), rabbit anti-phospho-Akt, serine 473 (Cell Signaling, 9271; 1:1,000), rabbit anti-Akt (Cell Signaling, 9272; 1:2,000), mouse anti-HIF1α (NovusBio, NBP100123; 1:500), rabbit anti-VEGF-A (Santa Cruz, SC152; 1:1,000). After overnight incubation, the membranes were probed with respective horseradish peroxidase (HRP)-conjugated secondary antibodies for one hour in room temperature: anti-goat (Santa Cruz, SC2354; 1:3,000), anti-rabbit (Sigma-Aldrich, A6154; 1:5,000) or recombinant mouse IgGκ light chain binding protein (Santa Cruz, SC516102; 1:3,000). The membranes were incubated with ECL substrate reagent (BioRad, 1705060) and the chemiluminescence signal was captured by Syngene Pxi imaging system (USA). The full blot for each antibody is shown in Supplementary data (**Additional File 1: Figure SVIIIA-J**).

The band intensity of protein of interest was normalized to the band intensity of a prominent band between 37 kDa and 50 kDa from Ponceau S stained blot (total protein expression level) [7, 8] and expressed as fold changes towards the control group [9]. This particular approach was taken because the protein expression of housekeeping genes such as α-tubulin and β-actin showed a decreasing trend in the *db/db* mice while the selected band between 37 kDa and 50 kDa (from Ponceau S stained blot) showed consistent expression in the ND, *db/db* and *db/db-ChAT-tg* mice (**Additional File 1: Figure SIX**). As for the quantification of the phosphorylation of Akt, the band intensity of the phosphorylated protein was normalized to total Akt expression as well as a prominent band between 37 kDa and 50 kDa on the Ponceau S stained blot. The rationale for the latter approach is that *(1)* total expression was significantly decreased in the *db/db* mice; *(2)* to further examine the phosphorylation status in a condition when the expression of normalizer is consistent among the ND, *db/db and db/db-ChAT-tg* mice. This particular approach has previously adopted by several research groups where they normalized to normalizer such as β-actin or GAPDH that showed consistent expression in their study[10, 11]. Although it is unknown why α-tubulin, β-actin and total Akt expression showed a decreasing trend, this could be due to enhanced protein degradation that is associated with diabetes mellitus[12-16].

Immunofluorescence analysis

The paraformaldehyde-fixed mouse ventricular tissues were coated in OCT and cut into seven micrometer thick section. For detection of ChAT, GLUT-4 and cardiac troponin I (CTNI, a cardiac

marker), triple-labelling immunofluorescence was performed on ND mouse ventricular tissues. Briefly, the sections were probed with anti-ChAT (Abcam, AB181023), anti-GLUT-4 (NovusBio, NBP1-49533) and biotin-conjugated anti-CTNI antibodies (NovusBio, NB110-2546B) in a sequential manner. DAPI (Santa Cruz, SC3598; 1:1,000) was then used to stain the nuclei. The specificity of the anti-ChAT antibody was tested on the ventricular tissue of *db/db-ChAT-tg* mice (served as a positive control; **Additional File 1: Figure SIIA**) while anti-CTNI antibody was tested on the skeletal muscle of ND mouse (served as negative control; **Additional File 1: Figure SIIB**). Besides, single staining of ChAT, GLUT-4 and CTNI on mouse ventricular tissues was individually performed to serve as a control to compare with the images obtained from triple-labelling staining (**Additional File 1: Figure SIIC)**

For microvascular analysis, the sections were probed with biotin-conjugated Isolectin-B4 (Vector laboratories, B1205; 1:200) and anti-α-smooth muscle actin conjugated with Cy3™ (Sigma-Aldrich, C6198; 1:800) to detect endothelial cells and smooth muscle cells, respectively. DAPI (Santa Cruz, SC3598; 1:1,000) was used to stain the nuclei. The density of capillaries was expressed as the mean number of $Isolectin^+$ $cells/mm^2$ of cardiac tissue. The density of small or large arterioles was expressed as the mean number of $\alpha SMA^+Isolectin^+$ $cells/mm^2$ of cardiac tissue.

Picrosirius staining

Seven microns thick cryosections were stained with 0.1% direct red 80 (Sigma Aldrich,3665548)/Picric acid solution (Sigma Aldrich, 197378) for one hour, and followed by washing in 0.5% acetic acid solution (Sigma Aldrich, 695092) and dehydrating in 50% ethanol, 85% ethanol, 95% ethanol and 100% ethanol sequentially for three minutes. The slides were mounted with DPX medium (Sigma-Aldrich, 06522) and dried overnight. The images were captured at 4X using an Olympus AX70 microscope with a polarizer. Image J software was used for image analysis. The fibrotic area was normalized to total tissue area and expressed as fold changes towards the control group.

In vitro cell culture experiments

Human ventricular cardiomyocyte cell line – AC16 cells

Human ventricular cardiomyocytes (AC16) cells were purchased from Davidson Laboratory at the University of Colombia in New York[17]. AC16 cells were maintained and grown in cell culture media composed of DMEM (Gibco, 31600034) with 5% FBS (Hyclone, SH30406.02) and 1X antibiotic-antimycotic solution (Gibco, 15240062), under 37ºC and 5% CO2. The complete DMEM medium was changed every two days.

Overexpression of human ChAT gene in AC16 cells

The AC16 cells were seeded at a density of $0.75x10^5$ $cells/cm^2$, which resulted in approximately 90% confluency after overnight attachment. Then, the cells were transfected with pReceiver-M83 expression plasmid with the human ChAT gene (M83-hChAT) or without an open reading frame (M83-neg) as a negative control for 24 hours. The expression plasmid allows simultaneous expression of mCherry fluorescent protein to determine the transfection efficiency.

Induction of insulin resistance in AC16 cells

The AC16 cells were seeded at a density of $0.6x10^5$ $cells/cm^2$, which resulted in approximately 70% confluency after overnight attachment. The cells were incubated in DMEM serum-free

medium containing 400 μM palmitate/4% BSA complex, 30 mM glucose, and 1X antibiotic-antimycotic solution for 48 hours (addressed as diabetic cells) to induce insulin resistance[18]. The AC16 cells which served as a vehicle control were incubated in DMEM serum-free medium containing 5.5 mM glucose, 25 mM mannitol (osmotic control), non-conjugated 4% BSA solution and 1X antibiotic-antimycotic solution for the same duration.

Western blot analysis for cell culture experiments

Total protein or cytosolic and membrane protein extraction from AC16 cells was performed. The cytosolic and membrane fraction of protein were separated as part of the experiments required to detect membrane-specific proteins. An equal amount of protein was loaded and electrophoresed and blotted for following protein targets, processed as described above. The protein expression of pan-cadherin (membrane protein) was detected via western blot analysis to confirm the successful separation of cytosolic and membrane protein (*data not shown*). The band intensity of the target protein was normalized to a prominent band between 37 kDa and 50 kDa on the Ponceau S stained blot as described earlier[19] and expressed as fold changes towards the control group.

Additional File 1: Figure Legends

Additional File 1: Figure SI. Protein expression changes in the diabetic human heart. A-C. Uncropped blots of the ChAT (**A**), CHT1 (**B**) and GLUT-4 (C) shown in figure 1A-C. **D-F.** Representative uncropped blots and quantitative bar graphs with scatter plots showing the protein expression of VAChT **(D)**, AChE **(E)** and M_2AChR **(F)** in the non-diabetic (ND)- and diabetic (D)-CAD patients. Data are presented as mean ± SEM. Unpaired T-test was performed. The number of samples per group is indicated in the figure.

Additional File 1: Figure SII. Examination of the specificity of antibodies used to detect ChAT, GLUT-4 and cTnI. Representative confocal images showing a strong fluorescent intensity of ChAT in the ventricular tissue of db/db-ChAT-tg mouse compared to that of the db/db mouse **(A)**; positive CTNI staining detected in the ventricular tissue of ND mouse while CTNI staining was negative in the skeletal muscle of ND mouse **(B)**; single staining of ChAT, GLUT-4 and CTNI on different ND ventricular tissues served as controls for comparison with the triple staining that performed on the same ND ventricular tissue **(C)**; secondary-antibody only control for triple staining (**D**).

Additional File 1: Figure SIII. Uncropped western blots of different cardiac NNCS components in the type 2 diabetic db/db mouse heart. Representative uncropped blots showing the protein expression of ChAT (**A**), M_2AChR **(B)**, AChE **(C)**, VAChT **(D)**, CHT1 **(E)**, and GLUT-4 **(F)** in the ND and diabetic db/db mice heart at early and established stage of diabetes.

Additional File 1: Figure SIV. A. Uncropped blots of ChAT shown in Figure 3A. **B-F.** Uncropped blots of M_2AChR (**B**), pAkT and Akt (**C**), HIF1α (**D**), GLUT-4 (**E**), VEGF (**F**) and TGF- β1 (**G**) shown in Figure 4A, 4B, 4C, 4D, 4F and 4G respectively.

Additional File 1: Figure SV. A. Box and whisker plots showing the diameter of the coronary vessels at baseline. **B**-**E**. The line graphs showing ACh **(B&C)** and SNP **(D&E)** in changing the diameter of coronary vessels. The percentage changes were sorted according to the coronary vessel size in the db/db and db/db-ChAT-tg mice at early and established stage of diabetes, respectively. Data are presented as mean ± SEM. A non-parametric Mann-Whitney U test was performed. The number of samples per group is indicated in the figure.

Additional File 1: Figure SVI. The arterioles density in the db/db-ChAT-tg mice. Representative immunofluorescence images and bar graphs with scatter plots showing the density of large and small arterioles as indicated by the number of αSMA- and Isolectin-stained smooth muscle cells in the myocardium of db/db and db/db-ChAT-tg mice heart at early and established stage of diabetes. Data are presented as mean ± SEM. A non-parametric Mann-Whitney U test was performed. Yellow arrows indicate large arterioles (>50 µm) while white arrows indicate small arterioles (<50 µm). The number of samples per group is indicated in the figure.

Additional File 1: Figure SVII. Diabetes reduced membrane GLUT-4 expression. A-C. Representative western blot images and quantitative scatter plot bar graph showing reduced pAkt expression (**A**), ChAT expression (**B**) and membrane GLUT-4 expression (**C**) in the diabetic human cardiomyocytes in response to insulin stimulation. Diabetic condition was induced by

treating the cells with high glucose (30mM) and palmitate (400μM). Reduced pAkt expression in response to insulin stimulation indicated the development of insulin resistance in these cells. Reduced membrane GLUT-4 expression suggests reduced glucose transport. ****p<0.0001 vs. corresponding no insulin stimulation group; #p<0.05, ##p<0.01, ###p<0.001 and ####p<0.0001 vs. corresponding control cardiomyocytes; $p<0.05 vs 30min insulin treatment group. **D.** Representative western blot images and quantitative scatter plot bar graph showing successful transfection with hChAT. ****p<0.0001 vs. wild type (WT) cardiomyocytes. **E.** Representative western blot images and quantitative scatter plot bar graph showing preserved membrane GLUT-4 expression in the diabetic cardiomyocytes overexpressed with hChAT. *p<0.05 and ****p<0.0001 vs. corresponding control; #p<0.05 vs. corresponding WT.

Additional File 1: Figure SVIII. Demonstration of the specificity of antibodies used in western blot. Representative full blots showing the specificity of anti-ChAT **(A)**, anti-M_2AChR **(B)**, anti-CHT1 **(C)**, anti-VAChT **(D)**, anti-AChE **(E)**, anti-GLUT-4 **(F)**, anti-VEGF-A **(G)**; anti-phosphorylated Akt at serine 473 **(H)**, anti-Akt **(I)** and anti-HIF1α antibodies **(J)** tested on protein lysates from various samples such as mouse brain tissue (positive control), AC16 cells, HL1 cells (mouse atrial cardiomyocytes cells), mouse ventricular tissues, human LV and right atrial appendage (RAA).

Additional File 1: Figure SIX. Protein expression of α-tubulin, β-actin and total protein from Ponceau S staining of the mouse ventricular tissues. A. Representative blots showing a decreasing trend in α-tubulin expression in the db/db mice at 8-weeks of age while β-actin expression and total protein as well as the selected band (red arrow) from Ponceau S staining showed consistent expression in all samples. **B & C.** Representative blot showing a decreasing trend in β-actin expression in the db/db mice at 16- and 20-weeks of age while total protein as well as selected band (red arrow) from Ponceau S staining showed relatively consistent expression in all samples.

Additional File 1: Figure SX: The role of cardiac NNCS in the diabetic heart. Summary of the proposed role for cardiac NNCS in the diabetic heart. VEGF-A – vascular endothelial growth factor; GLUT-4 – glucose transporter-4; HIF – hypoxia inducible factor; PI3K - Phosphoinositide 3-kinase.

References

1. Organization GWH. 2. Glycated haemoglobin (hba1c) for the diagnosis of diabetes. *Use of glycated haemoglobin (hba1c) in the diagnosis of diabetes mellitus: Abbreviated report of a who consultation*. 2011.
2. Rawal S, Munasinghe PE, Shindikar A, Paulin J, Cameron V, Manning P, Williams MJA, Jones GT, Bunton R, Galvin I, Katare R. Down-regulation of proangiogenic microrna-126 and microrna-132 are early modulators of diabetic cardiac microangiopathy. *Cardiovascular Research*. 2017;113:90-101
3. Rawal S, Nagesh PT, Coffey S, Van Hout I, Galvin IF, Bunton RW, Davis P, Williams MJA, Katare R. Early dysregulation of cardiac-specific microrna-208a is linked to maladaptive cardiac remodelling in diabetic myocardium. *Cardiovasc Diabetol*. 2019;18:13-13
4. Kakinuma Y, Tsuda M, Okazaki K, Akiyama T, Arikawa M, Noguchi T, Sato T. Heart-specific overexpression of choline acetyltransferase gene protects murine heart against ischemia through hypoxia-inducible factor-1α-related defense mechanisms. *J Am Heart Assoc*. 2013;2:e004887
5. Oikawa S, Kai Y, Tsuda M, Ohata H, Mano A, Mizoguchi N, Sugama S, Nemoto T, Suzuki K, Kurabayashi A, Muramoto K, Kaneda M, Kakinuma Y. Non-neuronal cardiac cholinergic system influences cns via the vagus nerve to acquire a stress-refractory propensity. *Clinical science (London, England : 1979)*. 2016;130:1913-1928
6. Katare R, Pearson JT, Lew JK-S, Wei M, Tsuchimouchi H, Du C-K, Zhan D-Y, Umetani K, Shirai M, Schwenke DO. Progressive decrease in coronary vascular function associated with type 2 diabetic heart disease. *Frontiers in physiology*. 2018;9:696-696
7. Rain S, Bos DdSG, Handoko ML, Westerhof N, Stienen G, Ottenheijm C, Goebel M, Dorfmüller P, Guignabert C, Humbert M, Bogaard H-J, Remedios CD, Saripalli C, Hidalgo CG, Granzier HL, Vonk-Noordegraaf A, van der Velden J, de Man FS. Protein changes contributing to right ventricular cardiomyocyte diastolic dysfunction in pulmonary arterial hypertension. *J Am Heart Assoc*. 2014;3:e000716-e000716
8. Pinto-Junior DC, Silva KS, Michalani ML, Yonamine CY, Esteves JV, Fabre NT, Thieme K, Catanozi S, Okamoto MM, Seraphim PM, Corrêa-Giannella ML, Passarelli M, Machado UF. Advanced glycation end products-induced insulin resistance involves repression of skeletal muscle glut4 expression. *Sci Rep*. 2018;8:8109
9. Katare R, Rawal S, Munasinghe PE, Tsuchimochi H, Inagaki T, Fujii Y, Dixit P, Umetani K, Kangawa K, Shirai M, Schwenke DO. Ghrelin promotes functional angiogenesis in a mouse model of critical limb ischemia through activation of proangiogenic micrornas. *Endocrinology*. 2016;157:432-445
10. Ishii M, Maeda A, Tani S, Akagawa M. Palmitate induces insulin resistance in human hepg2 hepatocytes by enhancing ubiquitination and proteasomal degradation of key insulin signaling molecules. *Arch Biochem Biophys*. 2015;566:26-35
11. Benzler M, Benzler J, Stoehr S, Hempp C, Rizwan MZ, Heyward P, Tups A. "Insulin-like" effects of palmitate compromise insulin signalling in hypothalamic neurons. *Journal of Comparative Physiology B*. 2019;189:413-424
12. Dice JF, Walker CD, Byrne B, Cardiel A. General characteristics of protein degradation in diabetes and starvation. *Proc. Natl. Acad. Sci. U.S.A.* 1978;75:2093-2097
13. Smith OL, Wong CY, Gelfand RA. Skeletal muscle proteolysis in rats with acute streptozocin-induced diabetes. *Diabetes*. 1989;38:1117-1122
14. Liu Z, Miers WR, Wei L, Barrett EJ. The ubiquitin-proteasome proteolytic pathway in heart vs skeletal muscle: Effects of acute diabetes. *Biochem. Biophys. Res. Commun.* 2000;276:1255-1260

15. Siew ED, Pupim LB, Majchrzak KM, Shintani A, Flakoll PJ, Ikizler TA. Insulin resistance is associated with skeletal muscle protein breakdown in non-diabetic chronic hemodialysis patients. *Kidney international*. 2007;71:146-152
16. Hu J, Klein JD, Du J, Wang XH. Cardiac muscle protein catabolism in diabetes mellitus: Activation of the ubiquitin-proteasome system by insulin deficiency. *Endocrinology*. 2008;149:5384-5390
17. Davidson MM, Nesti C, Palenzuela L, Walker WF, Hernandez E, Protas L, Hirano M, Isaac ND. Novel cell lines derived from adult human ventricular cardiomyocytes. *J Mol Cell Cardiol*. 2005;39:133-147
18. Chokshi A, Drosatos K, Cheema FH, Ji R, Khawaja T, Yu S, Kato T, Khan R, Takayama H, Knoll R, Milting H, Chung CS, Jorde U, Naka Y, Mancini DM, Goldberg IJ, Schulze PC. Ventricular assist device implantation corrects myocardial lipotoxicity, reverses insulin resistance, and normalizes cardiac metabolism in patients with advanced heart failure. *Circulation*. 2012;125:2844-2853
19. Rain S, Bos Dda S, Handoko ML, Westerhof N, Stienen G, Ottenheijm C, Goebel M, Dorfmuller P, Guignabert C, Humbert M, Bogaard HJ, Remedios CD, Saripalli C, Hidalgo CG, Granzier HL, Vonk-Noordegraaf A, van der Velden J, de Man FS. Protein changes contributing to right ventricular cardiomyocyte diastolic dysfunction in pulmonary arterial hypertension. *J Am Heart Assoc*. 2014;3:e000716

	ND-CAD (n=18)				D-CAD (n=10-12)							
	BMI		EF		BMI		HbA1c		Diabetes duration		EF	
	Pearson r	P value	Pearson r	P value	Pearson r	P value	Pearson r	P value	Pearson r	P value	Pearson r	P value
ChAT	0.1715	0.5	0.05566	0.83	-0.437	0.18	-0.4592	0.15	-0.1152	0.74	-0.2807	0.43
CHT1	0.5814	0.01*	-0.001781	0.99	-0.3206	0.34	-0.3144	0.35	0.4759	0.14	-0.3611	0.31
VAChT	-0.1766	0.48	0.209	0.41	0.3101	0.35	-0.2662	0.43	-0.1402	0.68	0.02249	0.95
AChE	0.4289	0.08	0.1793	0.48	-0.4144	0.18	-0.09356	0.77	-0.2348	0.46	-0.2771	0.41
M2AChR	-0.1301	0.61	-0.07385	0.77	0.4137	0.18	-0.01248	0.97	-0.09574	0.77	-0.08418	0.81
GLUT4	0.2097	0.40	-0.2997	0.23	-0.2411	0.45	-0.2007	0.53	0.4148	0.18	-0.4689	0.15

Additional File 1: Table S1: Pearson correlation analysis showing correlation between NNCS components and clinical parameters. BMI – body mass index; EF – ejection fraction; HbA1c – glycated haemoglobin.

Additional File: Figure SI

Western blotting uncropped figures - Main Figure 1A-C

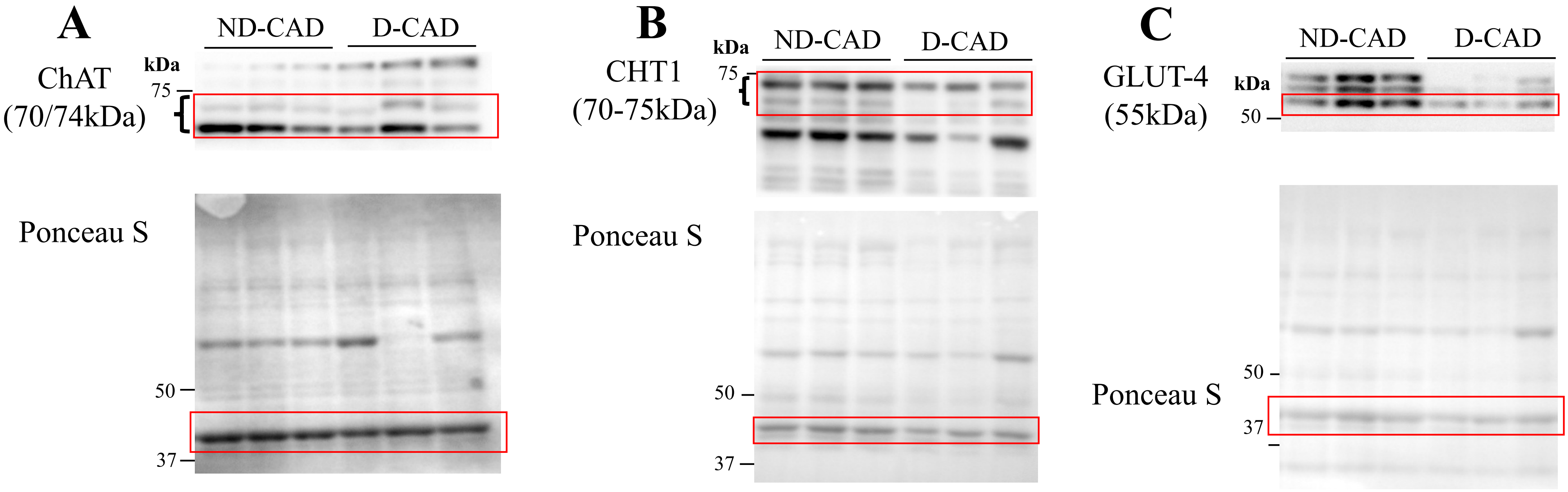

Additional File: Figure SI

Western blotting uncropped figures

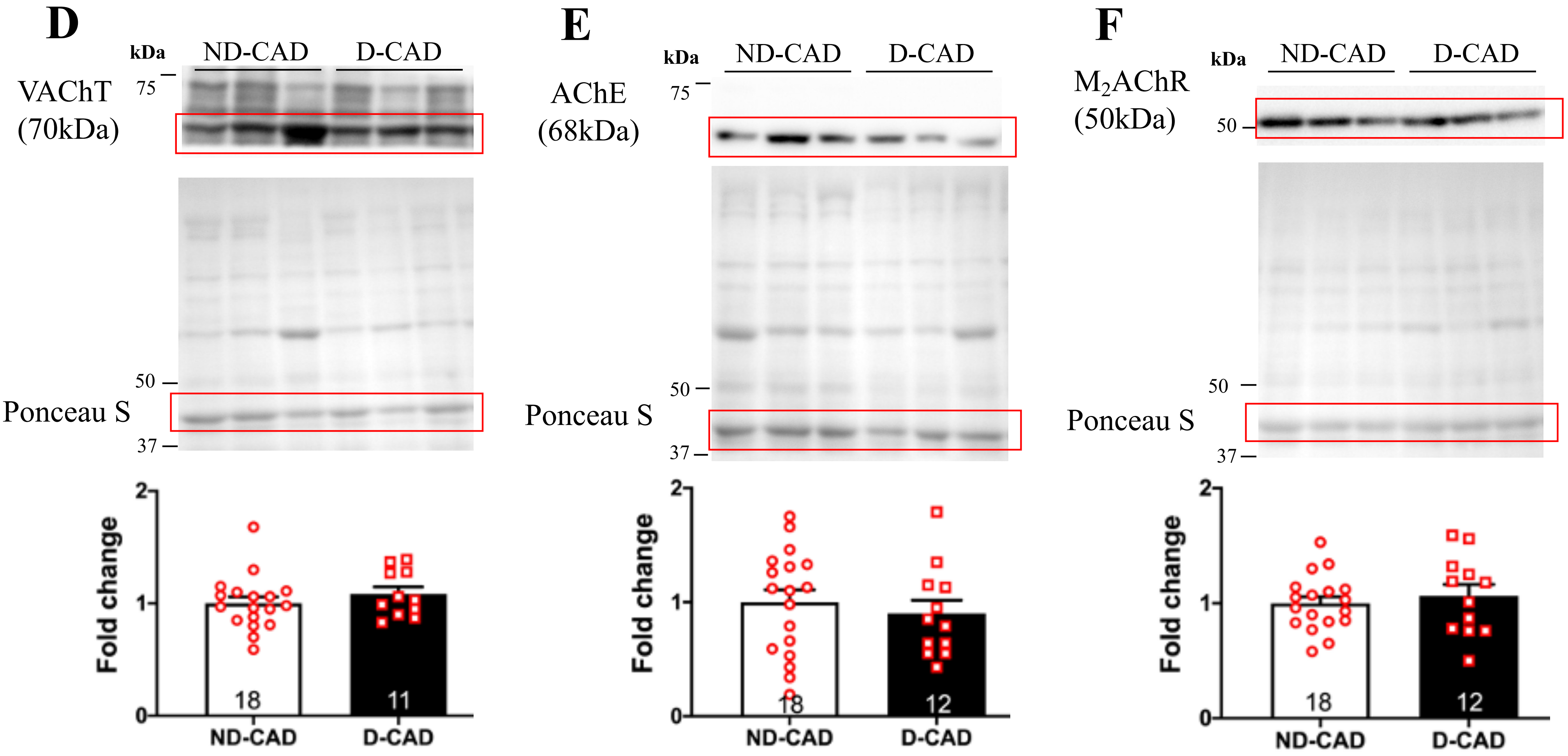

Online Figure I. Protein expression changes in the diabetic human heart. A-C. Uncropped blots of the ChAT (**A**), CHT1 (**B**) and GLUT-4 (C) shown in figure 1A-C. **D-F.** Representative uncropped blots and quantitative bar graphs with scatter plots showing the protein expression of VAChT **(D)**, AChE **(E)** and M_2AChR **(F)** in the non-diabetic (ND)- and diabetic (D)-CAD patients. Data are presented as mean ± SEM. Unpaired T-test was performed. The number of samples per group is indicated in the figure.

Additional File: Figure SII

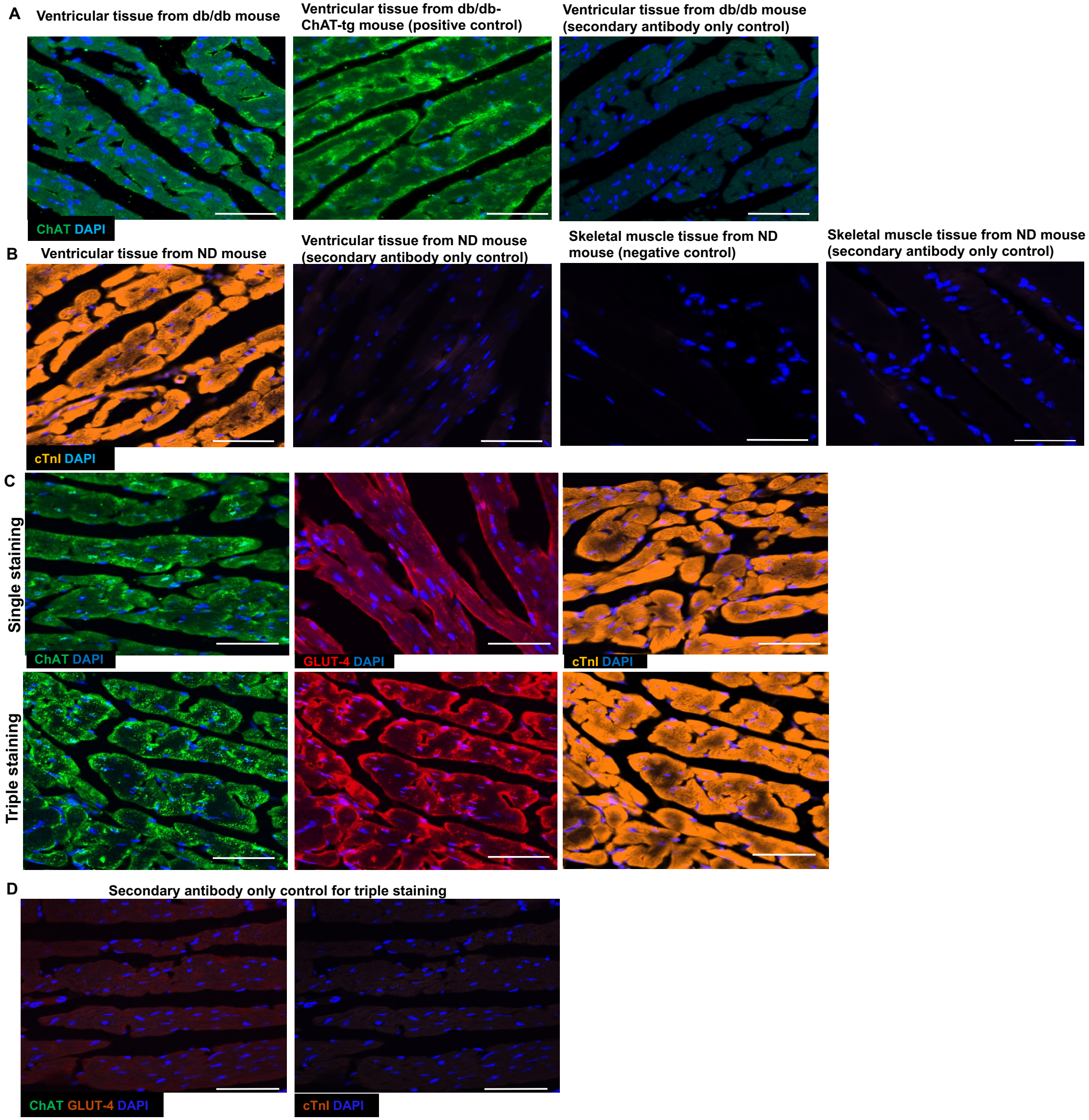

Online Figure II. Examination of the specificity of antibodies used to detect ChAT, GLUT-4 and cTnI. Representative confocal images showing a strong fluorescent intensity of ChAT in the ventricular tissue of db/db-ChAT-tg mouse compared to that of the db/db mouse **(A)**; positive CTNI staining detected in the ventricular tissue of ND mouse while CTNI staining was negative in the skeletal muscle of ND mouse **(B)**; single staining of ChAT, GLUT-4 and CTNI on different ND ventricular tissues served as controls for comparison with the triple staining that performed on the same ND ventricular tissue **(C)**; secondary-antibody only control for triple staining (**D**).

Additional File: Figure SIII

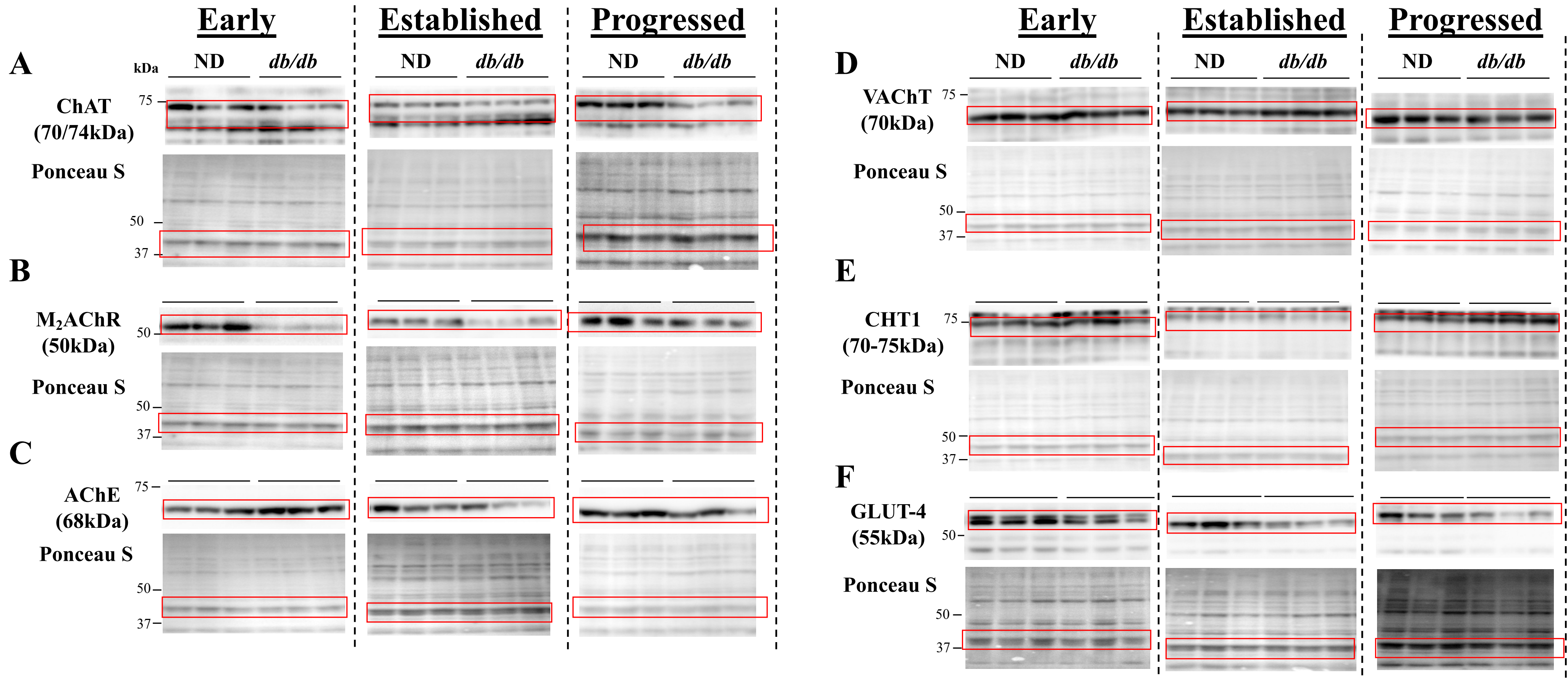

Online Figure III. Uncropped western blots of different cardiac NNCS components in the type 2 diabetic db/db mouse heart. Representative uncropped blots showing the protein expression of ChAT (**A**), M_2AChR **(B)**, AChE **(C)**, VAChT **(D)**, CHT1 **(E)**, and GLUT-4 **(F)** in the ND and diabetic db/db mice heart at early and established stage of diabetes.

Additional File: Figure SIV

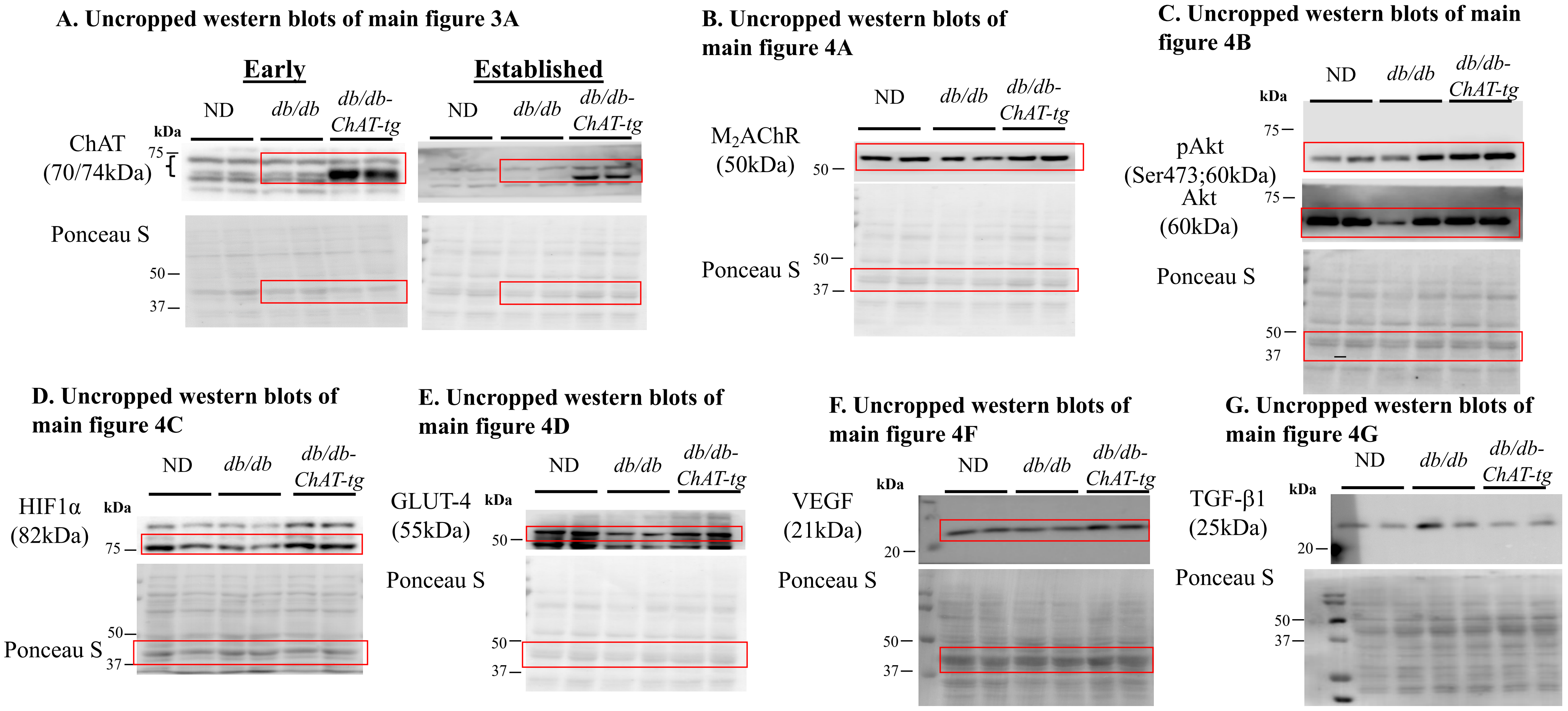

Online Figure IV. A. Uncropped blots of ChAT shown in Figure 3A. **B-F.** Uncropped blots of M_2AChR (**B**), pAkT and Akt (**C**), HIF1α (**D**), GLUT-4 (**E**), VEGF (**F**) and TGF- β1 (**G**) shown in Figure 4A, 4B, 4C, 4D, 4F and 4G respectively.

Additional File: Figure SV

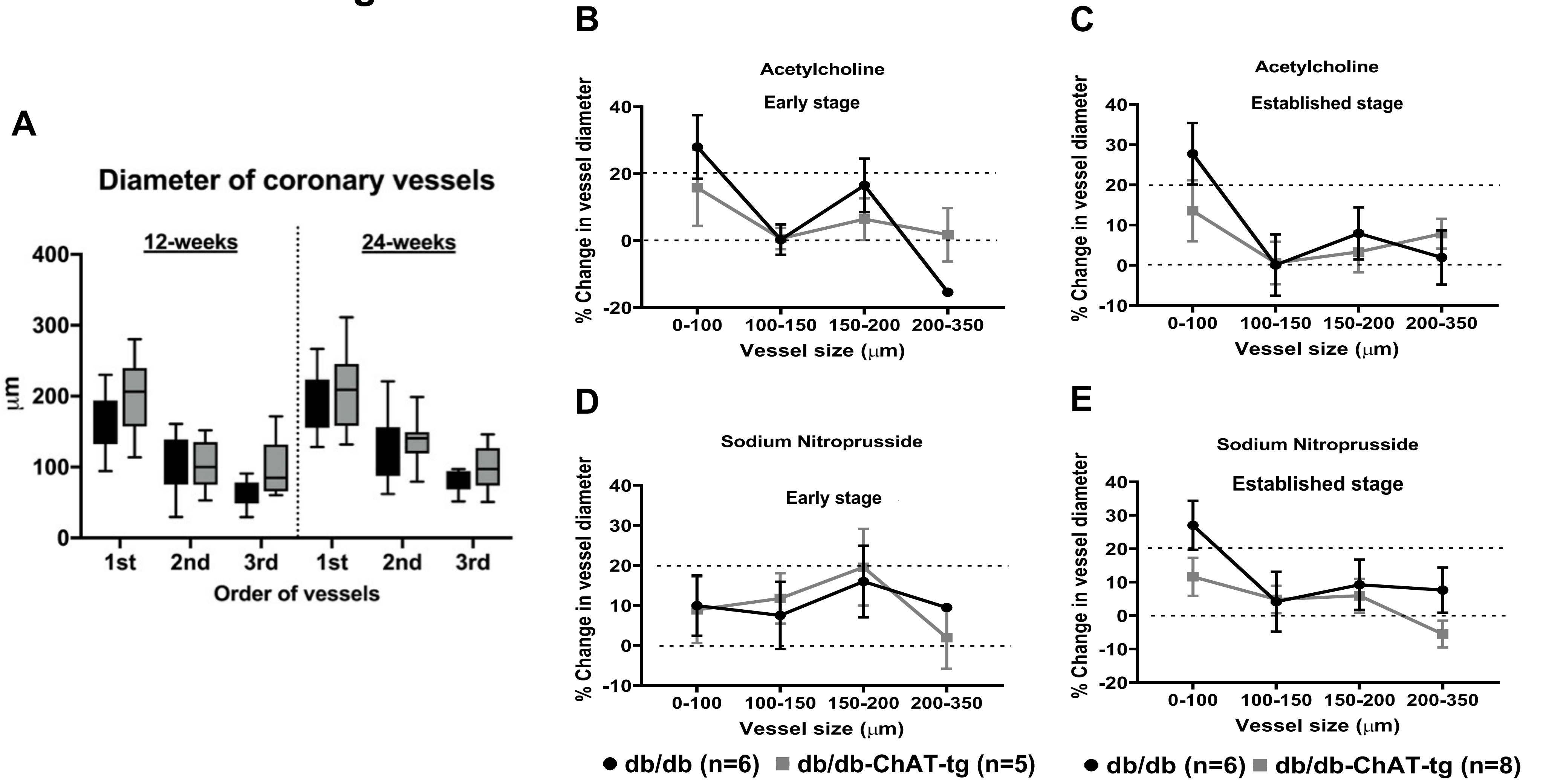

Online Figure V. A. Box and whisker plots showing the diameter of the coronary vessels at baseline. **B-E**. The line graphs showing ACh **(B&C)** and SNP **(D&E)** in changing the diameter of coronary vessels. The percentage changes were sorted according to the coronary vessel size in the db/db and db/db-ChAT-tg mice at early and established stage of diabetes, respectively. Data are presented as mean ± SEM. A non-parametric Mann-Whitney U test was performed. The number of samples per group is indicated in the figure.

Additional File: Figure SVI

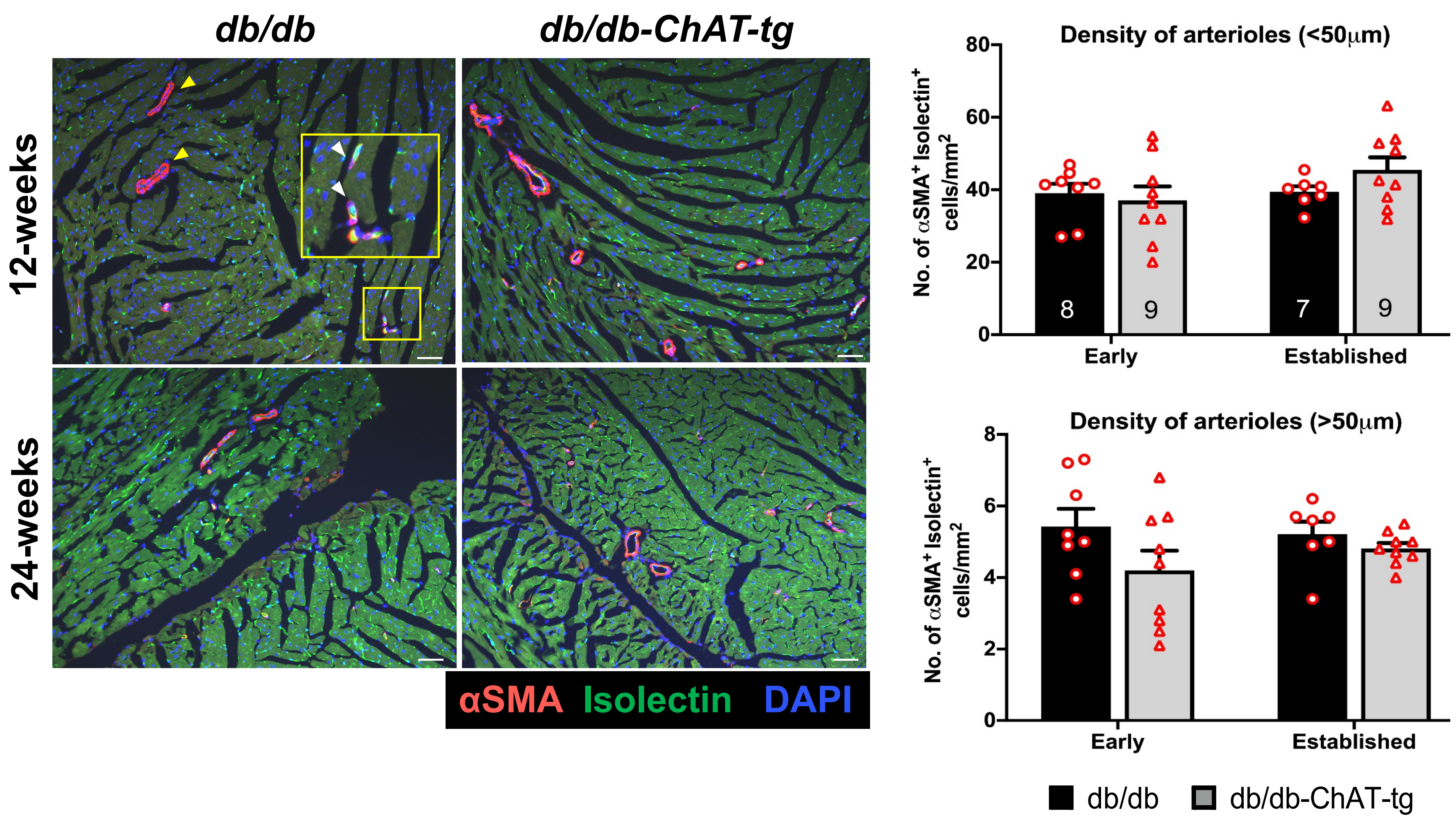

Online Figure VI. The arterioles density in the db/db-ChAT-tg mice. Representative immunofluorescence images and bar graphs with scatter plots showing the density of large and small arterioles as indicated by the number of αSMA- and Isolectin-stained smooth muscle cells in the myocardium of db/db and db/db-ChAT-tg mice heart at early and established stage of diabetes. Data are presented as mean ± SEM. A non-parametric Mann-Whitney U test was performed. Yellow arrows indicate large arterioles (>50 μm) while white arrows indicate small arterioles (<50 μm). The number of samples per group is indicated in the figure.

Additional File: Figure SVII

A. In vitro diabetic model

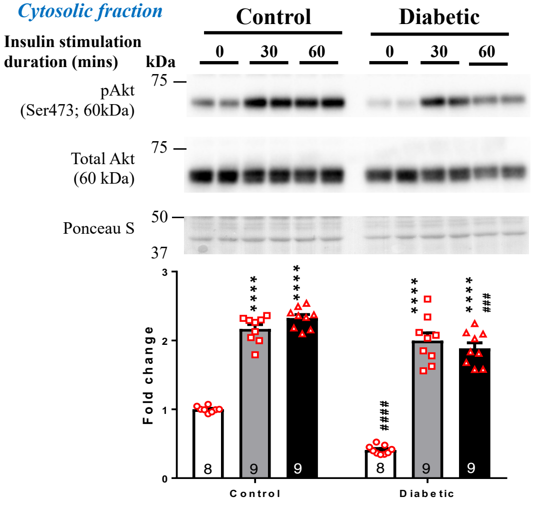

B. Reduced ChAT expression in the diabetic cardiomyocytes

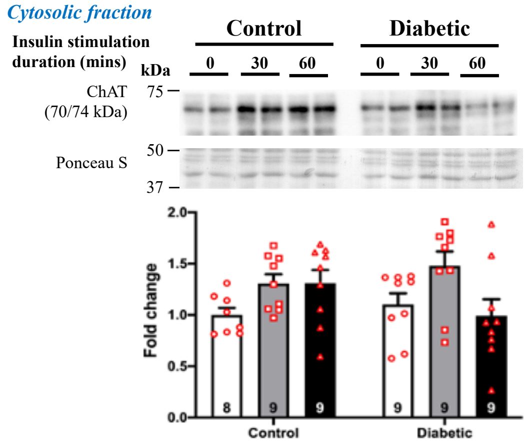

C. Reduced membrane GLUT-4 protein expression following insulin stimulation in diabetic cardiomyocytes

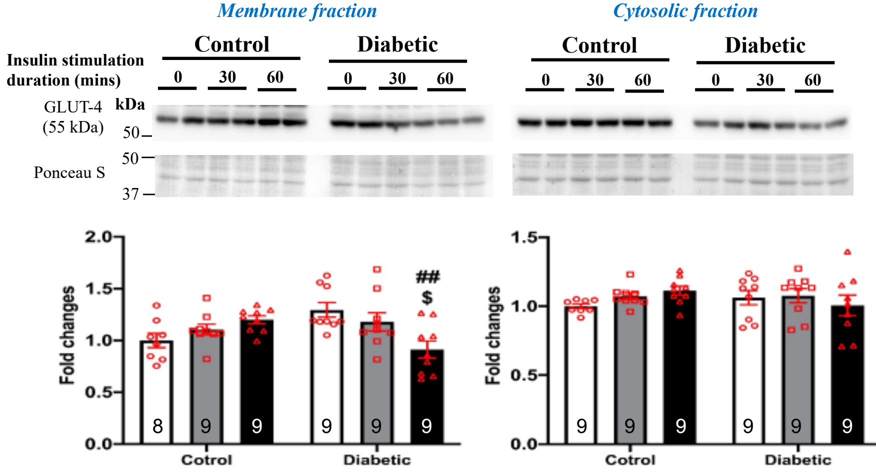

D. Successful transfection of ChAT in human cardiomyocytes

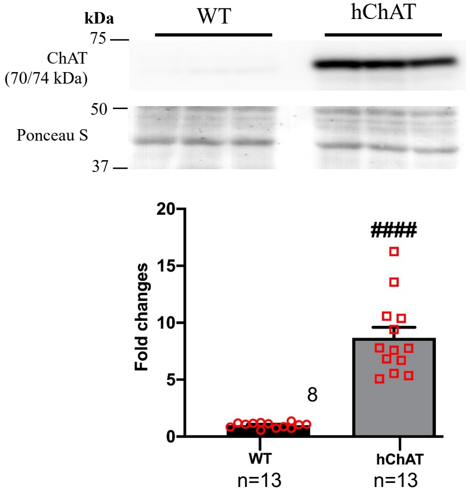

E. Overexpression of hChAT preserved membrane GLUT-4 protein expression in diabetic cardiomyocytes

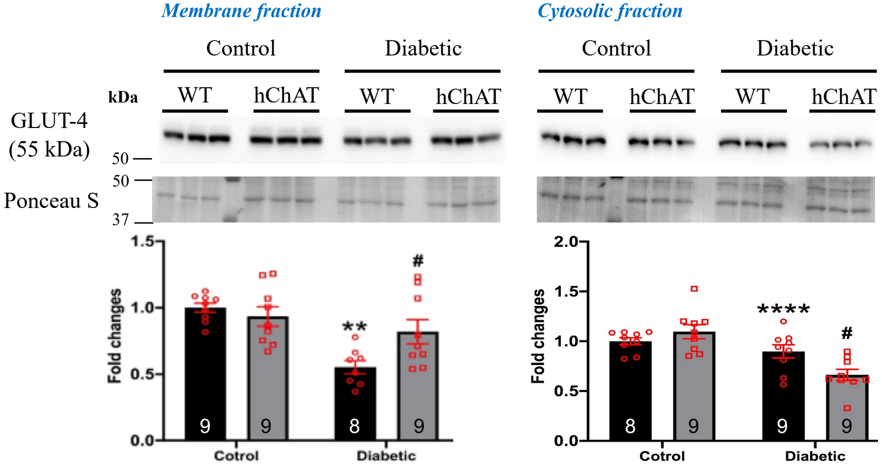

Online Figure VII. Diabetes reduced membrane GLUT-4 expression. A-C. Representative western blot images and quantitative scatter plot bar graph showing reduced pAkt expression (**A**), ChAT expression (**B**) and membrane GLUT-4 expression (**C**) in the diabetic human cardiomyocytes in response to insulin stimulation. Diabetic condition was induced by treating the cells with high glucose (30mM) and palmitate (400mM). Reduced pAkt expression in response to insulin stimulation indicated the development of insulin resistance in these cells. Reduced membrane GLUT-4 expression suggests reduced glucose transport. ****p<0.0001 vs. corresponding no insulin stimulation group; #p<0.05, ##p<0.01, ###p<0.001 and ####p<0.0001 vs. corresponding control cardiomyocytes; $p<0.05 vs 30min insulin treatment group. **D.** Representative western blot images and quantitative scatter plot bar graph showing successful transfection with hChAT. ****p<0.0001 vs. wild type (WT) cardiomyocytes. **E.** Representative western blot images and quantitative scatter plot bar graph showing preserved membrane GLUT-4 expression in the diabetic cardiomyocytes overexpressed with hChAT. *p<0.05 and ****p<0.0001 vs. corresponding control; #p<0.05 vs. corresponding WT.

Additional File: Figure SVIII

A. Anti-ChAT antibody

Chemicon, AB144P, predicted 70/74 kDa
UniProt database: 71,853 Da

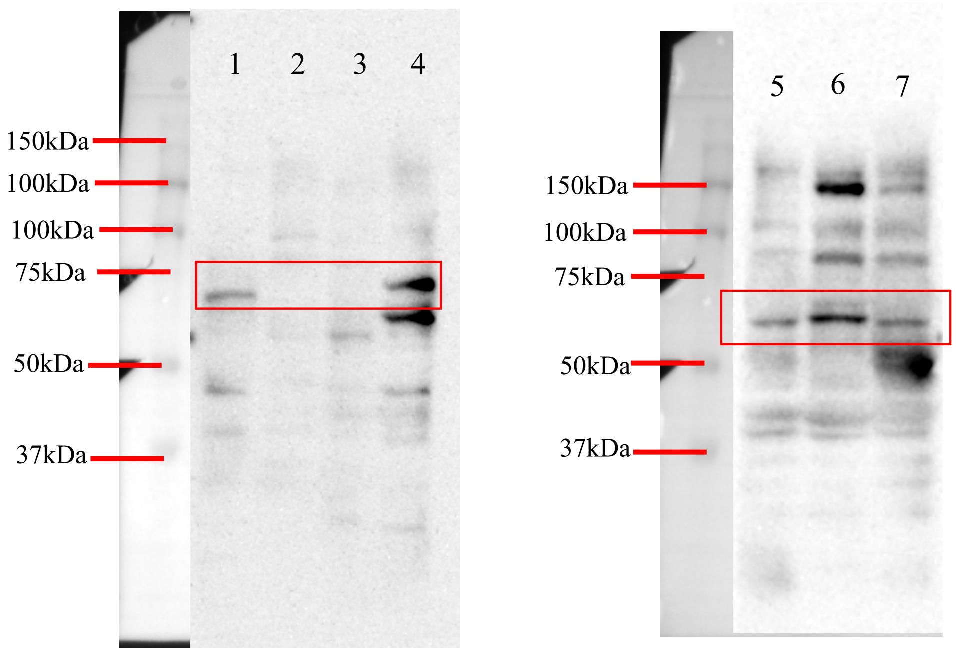

B. Anti-M_2AChR antibody

Abcam, AB109226, predicted 52 kDa
UniProt database: 51,715 Da

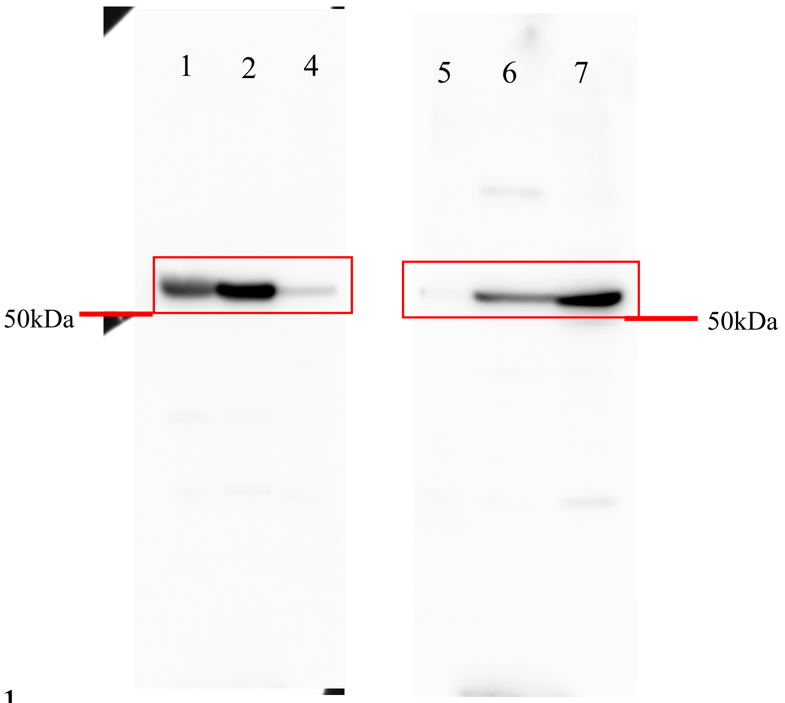

1: Brain tissues from 8-weeks ND mouse
2: Human ventricular AC16 cells
3: Mouse atrial HL1 cells
4: Ventricular tissues from 8-weeks ND mouse

5: Human LV-1
6: Human LV-2
7: Human RAA

C. Anti-CHT1 antibody

Merck Millipore, ABN458 predicted 70-75 kDa
UniProt database: 63,204 Da (+ 5 kDa due to glycosylation)

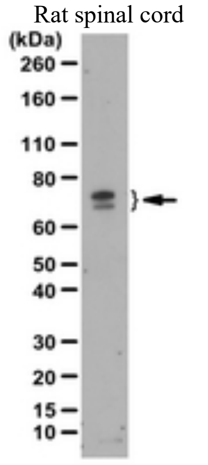

Blot obtained from Merck Millipore

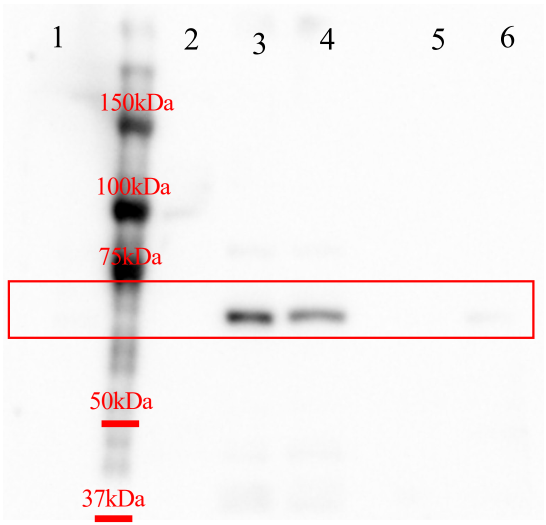

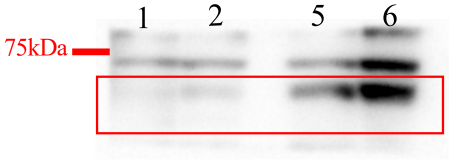

D. Anti-VAChT antibody

Sigma, SAB4200560 predicted 70 kDa
UniProt database: 56,903Da

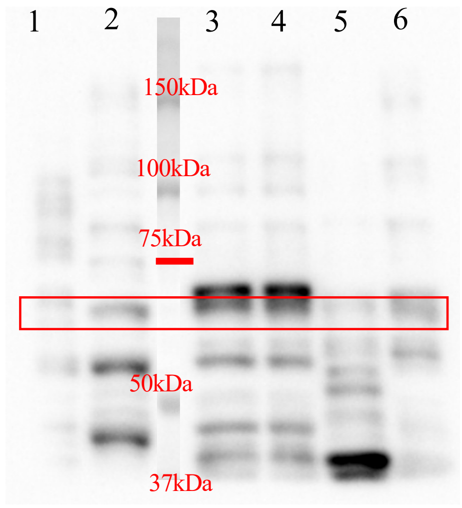

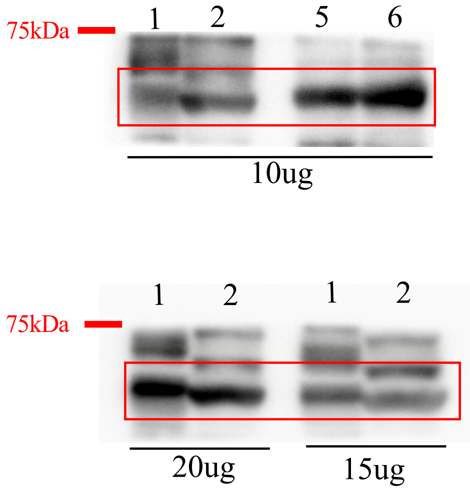

1: Brain tissues from 8-weeks ND mouse
2: Human ventricular AC16 cells
3: Ventricular tissues from 8-weeks ND mouse
4: Ventricular tissues from 16-weeks ND mouse
5: Human RAA
6: Human LV

E. Anti-AChE antibody

Bioss BS-2511R, predicted 68 kDa
UniProt database: 67,796 Da

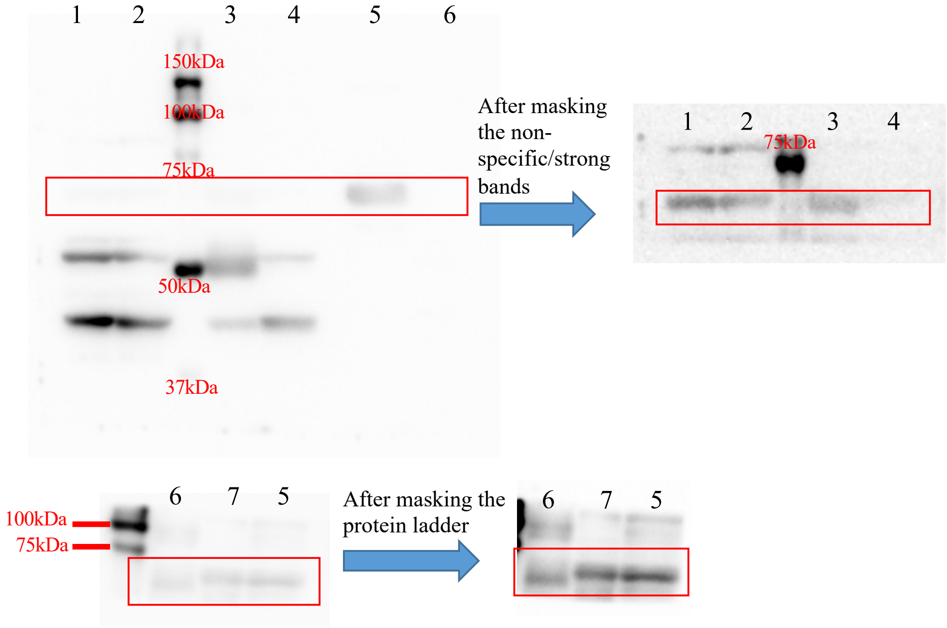

F. Anti-GLUT4 antibody

NovusBio, predicted 55 kDa, validated by biological strategies (by positive and negative expressing cells)
UniProt database: 54,787 Da

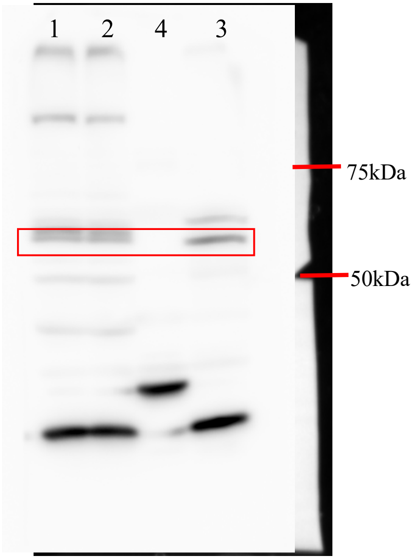

G. Anti- VEGF-A antibody

Santa cruz, predicted 21 kDa
UniProt database: 22,440 Da

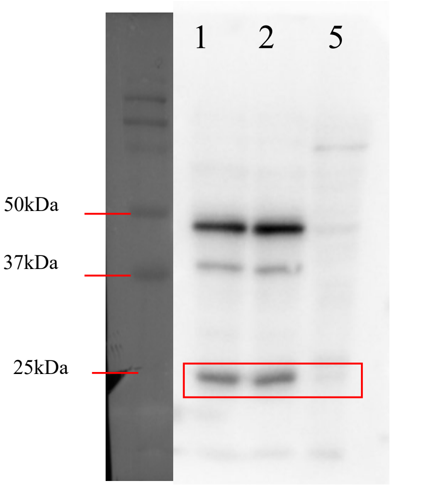

1: Ventricular tissues from 8-weeks ND mouse
2: Ventricular tissues from 16-weeks ND mouse
3: Human RAA
4: Human LV
5: Human ventricular AC16 cells
6: Brain tissues from 8-weeks ND mouse
7: Mouse atrial HL1 cells

H. Anti-phosphorylated Akt antibody (serine 473)
Cell signaling, predicted 60 kDa
UniProt database: 55,707 Da

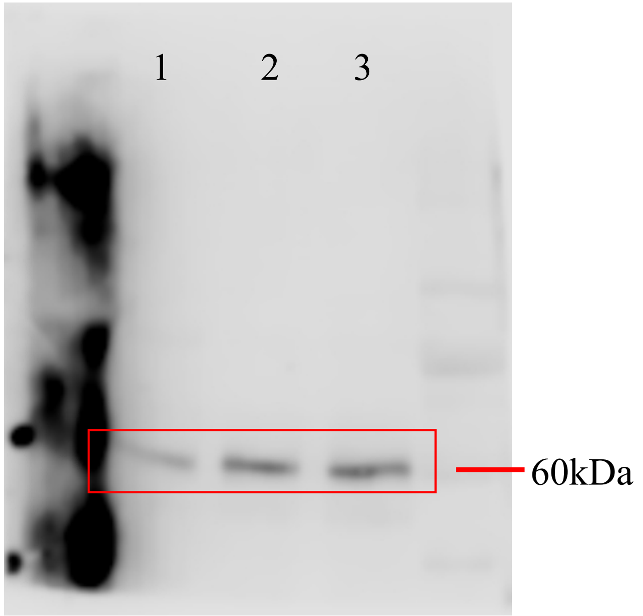

I. Anti-Akt antibody
Cell signaling, predicted 60 kDa
UniProt database: 55,707 Da

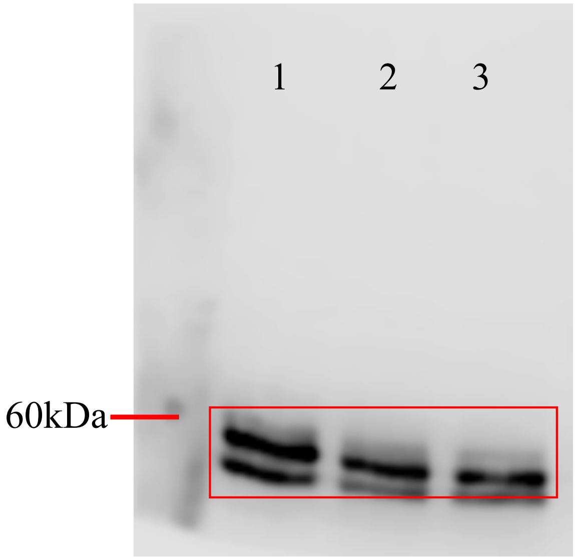

J. Anti-HIF1α antibody
Cell signaling, predicted 93 kDa
UniProt database: 92,670 Da (Isoform1) & 82,746 Da (Isoform2) & 95,634 Da (Isoform3)

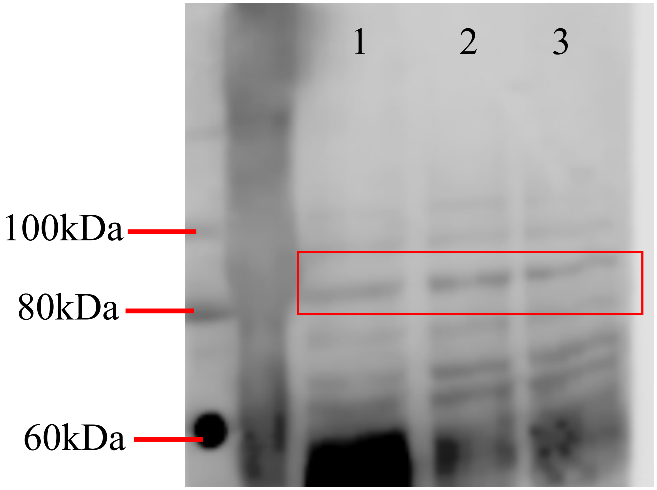

1: ventricular tissues from ND mouse
2: ventricular tissue from *db/db* mouse
3: ventricular tissue from *db/db-ChAT-tg* mouse

Online Figure VIII. Demonstration of the specificity of antibodies used in western blot. Representative full blots showing the specificity of anti-ChAT **(A)**, anti-M_2AChR **(B)**, anti-CHT1 **(C)**, anti-VAChT **(D)**, anti-AChE **(E)**, anti-GLUT-4 **(F)**, anti-VEGF-A **(G)**; anti-phosphorylated Akt at serine 473 **(H)**, anti-Akt **(I)** and anti-HIF1α antibodies **(J)** tested on protein lysates from various samples such as mouse brain tissue (positive control), AC16 cells, HL1 cells (mouse atrial cardiomyocytes cells), mouse ventricular tissues, human LV and right atrial appendage (RAA).

Additional File: Figure SIX

Expression of α-tubulin and β-actin is decreased in *db/db* mice

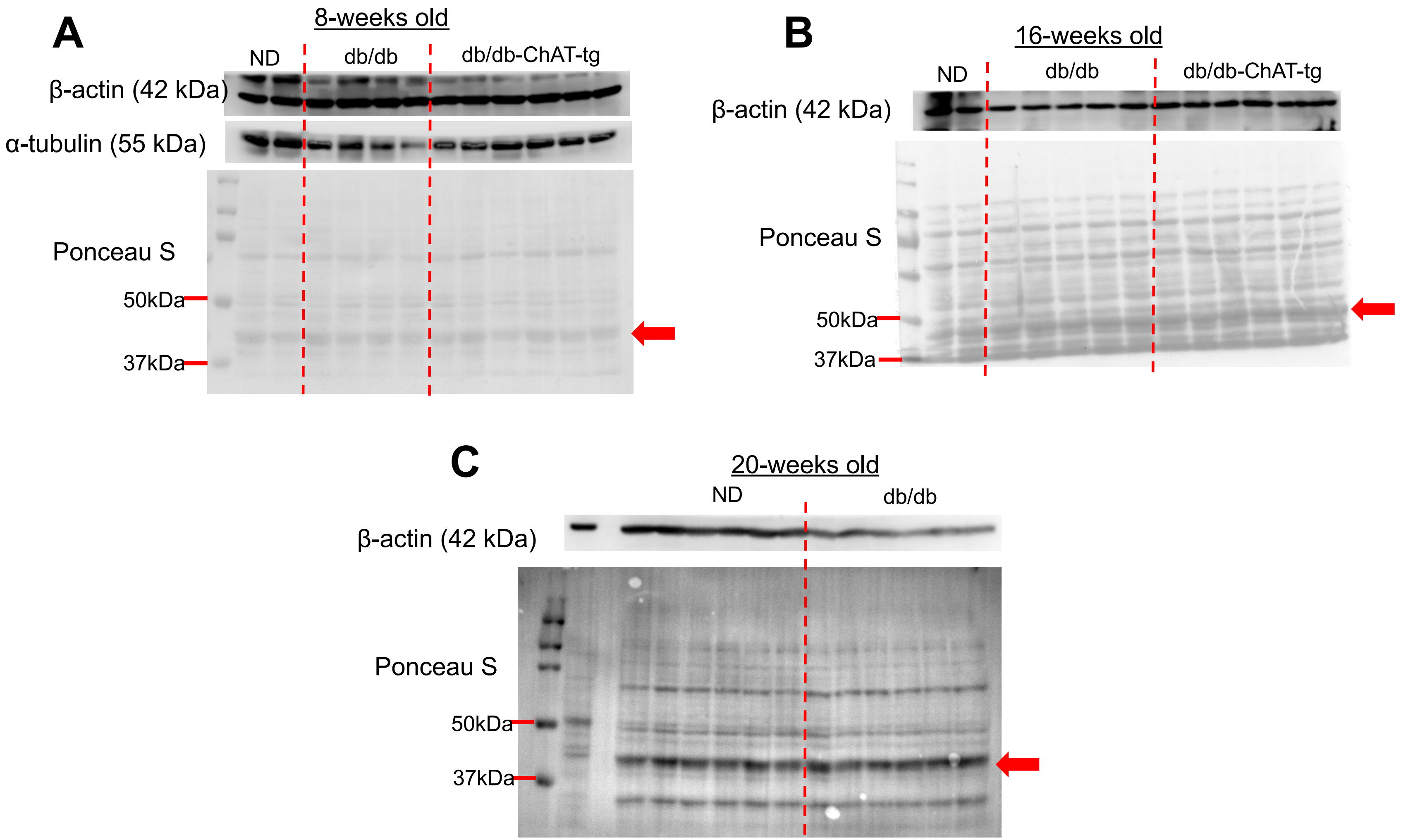

Online Figure IX. Protein expression of α-tubulin, β-actin and total protein from Ponceau S staining of the mouse ventricular tissues. A. Representative blots showing a decreasing trend in α-tubulin expression in the db/db mice at 8-weeks of age while β-actin expression and total protein as well as the selected band (red arrow) from Ponceau S staining showed consistent expression in all samples. **B & C.** Representative blot showing a decreasing trend in β-actin expression in the db/db mice at 16- and 20-weeks of age while total protein as well as selected band (red arrow) from Ponceau S staining showed relatively consistent expression in all samples.

Additional File: Figure SX - The role of cardiac NNCS in diabetic heart

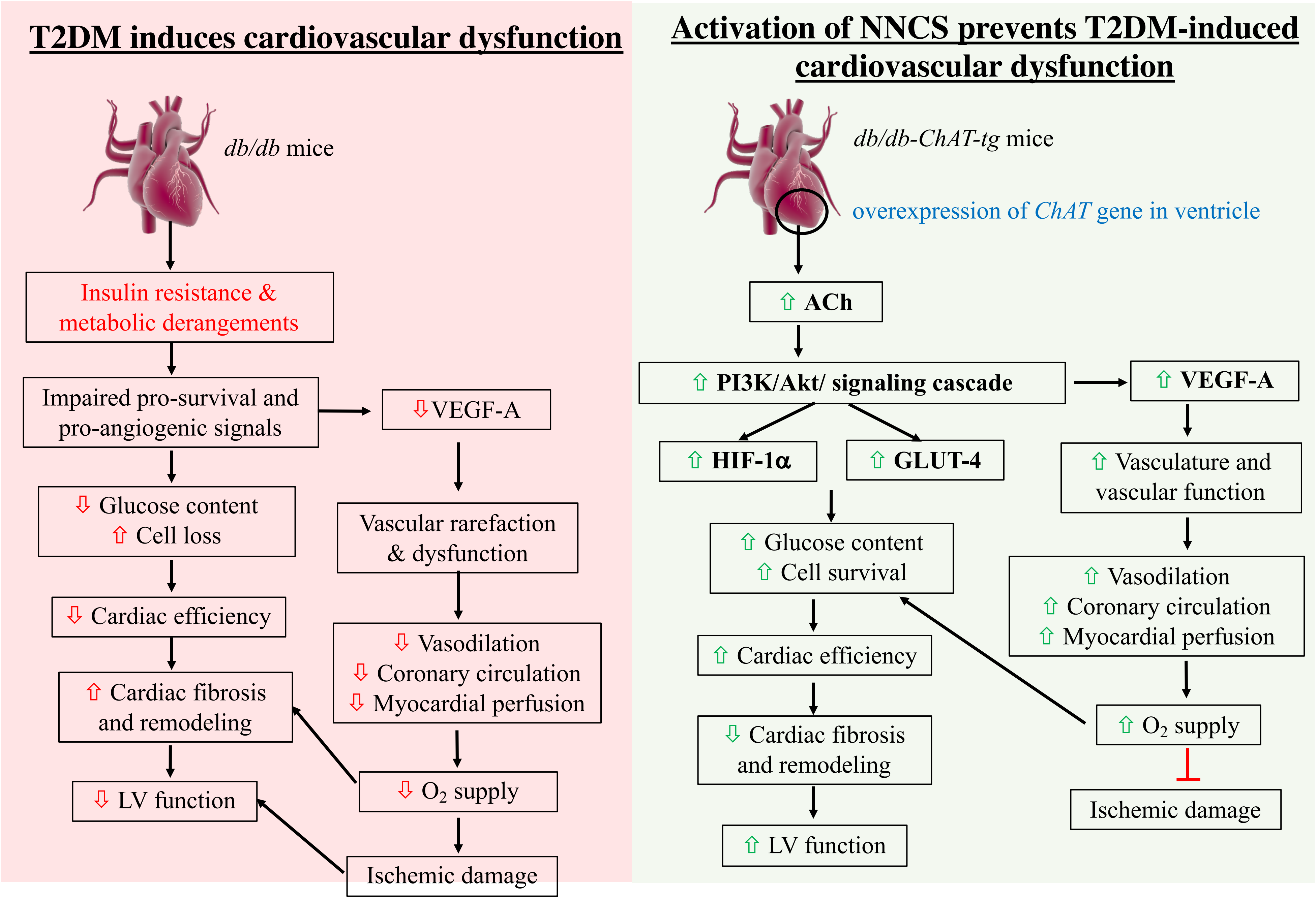

Supplement: Supplementary file 1 — Additional file 1. Expanded methods and additional figures. [file 12933_2021_1231_MOESM1_ESM.pdf]
